# Ischemic Heart Disease: Noninvasive Imaging Techniques and Findings

**DOI:** 10.1148/rg.2021200125

**Published:** 2021-05-21

**Authors:** Arlene Sirajuddin, S. Mojdeh Mirmomen, Seth J. Kligerman, Daniel W. Groves, Allen P. Burke, Faraz Kureshi, Charles S. White, Andrew E. Arai

**Affiliations:** From the Cardiovascular and Pulmonary Branch, National Heart Lung and Blood Institute, National Institutes of Health, 10 Center Dr, Building 10, Room B1D416, Bethesda, MD 20814 (A.S., S.M.M., A.E.A.); Department of Radiology, University of California San Diego, San Diego, Calif (S.J.K.); Departments of Medicine and Radiology, Divisions of Cardiology and Cardiothoracic Imaging, University of Colorado Anschutz Medical Campus, Aurora, Colo (D.W.G.); Department of Pathology (A.P.B.) and Department of Radiology and Nuclear Medicine (C.S.W.), School of Medicine, University of Maryland, Baltimore, Md; and St David’s Healthcare and Austin Heart, Austin, Tex (F.K.).

## Abstract

Ischemic heart disease is a leading cause of death worldwide and comprises a large proportion of annual health care expenditure. Management of ischemic heart disease is now best guided by the physiologic significance of coronary artery stenosis. Invasive coronary angiography is the standard for diagnosing coronary artery stenosis. However, it is expensive and has risks including vascular access site complications and contrast material–induced nephropathy. Invasive coronary angiography requires fractional flow reserve (FFR) measurement to determine the physiologic significance of a coronary artery stenosis. Multiple noninvasive cardiac imaging modalities can also anatomically delineate or functionally assess for significant coronary artery stenosis, as well as detect the presence of myocardial infarction (MI). While coronary CT angiography can help assess the degree of anatomic stenosis, its inability to assess the physiologic significance of lesions limits its specificity. Physiologic significance of coronary artery stenosis can be determined by cardiac MR vasodilator or dobutamine stress imaging, CT stress perfusion imaging, FFR CT, PET myocardial perfusion imaging (MPI), SPECT MPI, and stress echocardiography. Clinically unrecognized MI, another clear indicator of physiologically significant coronary artery disease, is relatively common and is best evaluated with cardiac MRI. The authors illustrate the spectrum of imaging findings of ischemic heart disease (coronary artery disease, myocardial ischemia, and MI); highlight the advantages and disadvantages of the various noninvasive imaging methods used to assess ischemic heart disease, as illustrated by recent clinical trials; and summarize current indications and contraindications for noninvasive imaging techniques for detection of ischemic heart disease.

Online supplemental material is available for this article.

Published under a CC BY 4.0 license.

## Graphical abstract

**Figure:**
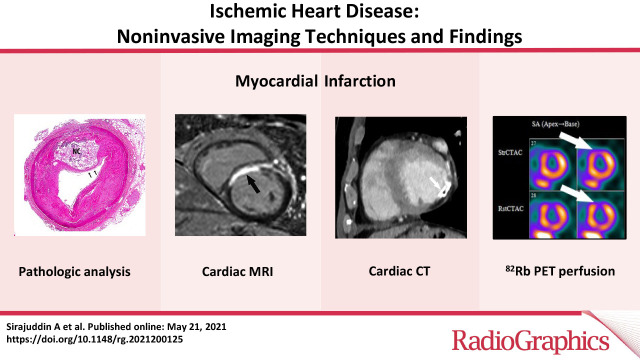

## SA-CME LEARNING OBJECTIVES

*After completing this journal-based SA-CME activity, participants will be able to:*

■ Describe how various noninvasive imaging methods help assess for myocardial ischemia, and list the advantages and disadvantages of each method.
■ Recognize the appearance of MI at all of the various noninvasive imaging modalities.
■ Recognize the imaging findings of complications related to MI.

## Introduction

There are currently about 126.5 million cases of ischemic heart disease in the world. Ischemic heart disease remains one of the leading causes of death, accounting for over 9 million deaths per year worldwide (1,2). According to the American Heart Association, the annual incidence of new coronary events is approximately 720 000 cases per year, and the current prevalence of ischemic heart disease is approximately 18.2 million cases in the United States alone (2). Invasive coronary angiography is considered the standard for detecting epicardial coronary artery stenoses, but it is expensive, carries some risk, and cannot assess the physiologic impact of a stenosis unless direct fractional flow reserve (FFR) measurements are obtained. Anatomically, 50% or more left main artery stenosis or 70% or more stenosis in any of the other coronary arteries is considered severe (3). However, the severity of a coronary artery stenosis does not always correlate with its hemodynamic significance. A stenosis greater than or equal to 70% sometimes may not lead to flow limitation, while two-thirds of moderate stenoses (50%–69% stenosis) are not flow limiting (4). In addition, misclassification of intermediate stenosis severity frequently occurs.

To better delineate the physiologic significance of a coronary artery stenosis, invasive FFR measurement can be performed during invasive coronary angiography. Invasive FFR is the ratio of coronary artery pressure distal to a stenosis to the aortic pressure during maximal hyperemia, with a ratio less than or equal to 0.8, representing a 20% decrease in pressure across a stenosis, being considered hemodynamically significant (3,5,6). Invasive FFR measurement requires passing a coronary pressure guidewire across the coronary stenosis to measure the pressure distal to the coronary stenosis during the intravenous administration of a vasodilator agent such as adenosine. Overall, invasive FFR measurement is a safe procedure. However, owing to its invasive nature, there is a small (<1%) risk of coronary artery injury (7). Noninvasive cardiac imaging modalities are also available to anatomically delineate or functionally assess for significant coronary artery stenosis, as well as detect the presence of myocardial infarction (MI).

Coronary CT angiography (CCTA) accurately detects coronary artery stenosis and grades the degree of stenosis on the basis of the percentage of luminal narrowing. However, additional evaluation to assess the physiologic significance of the coronary stenosis is often required. Multiple imaging techniques can help in the detection of the physiologic significance of coronary artery stenoses: cardiac MR vasodilator or dobutamine stress imaging, CT stress perfusion imaging, FFR CT, PET myocardial perfusion imaging (MPI), SPECT MPI, and stress echocardiography. In the past several years, significant advances in noninvasive imaging techniques now allow accurate noninvasive disease assessment that is equivalent to that of invasive coronary angiography and invasive FFR measurement. MI is best evaluated with cardiac MRI. Clinically unrecognized MI, which is another sign of physiologically significant coronary artery disease (CAD), is a relatively common finding that has been found in approximately 17% of older individuals (age ≥68 years) (8–10).

In this article, we summarize current indications and contraindications for noninvasive imaging techniques for detection of ischemic heart disease secondary to epicardial CAD, illustrate the spectrum of imaging findings of ischemic heart disease (CAD, myocardial ischemia, and MI including its complications), and highlight the advantages and disadvantages of the various noninvasive imaging methods used to assess ischemic heart disease, as illustrated by recent clinical trials.

## Pathophysiology of Ischemic Heart Disease

Coronary artery atherosclerotic plaque results from a complex process that is driven by multiple factors within the intimal layer of the coronary artery wall. These include endothelial cell dysfunction, oxidized serum lipids, inflammation, and thrombosis, with secondary effects of angiogenesis and calcification. These factors are largely mirrored by CAD risk factors, including dyslipidemia, obesity, elevated serum inflammatory marker levels, and hypertension (11,12). While imaging has much lower resolution than that of microscopic pathologic evaluation, noninvasive imaging can detect many pathophysiologic processes detailed in this section that are important to ischemic heart disease.

**Figure 1.**
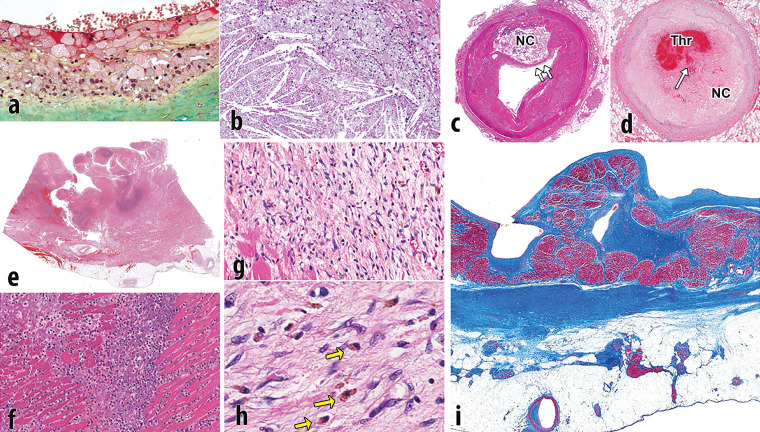
Pathophysiology of atherosclerotic plaque and MI. **(a)** Photomicrograph shows the surface of intimal plaque, with a luminal layer of foamy macrophages. The proteoglycan-rich fibrous portion of the plaque is depicted below (green area) (Movat pentachrome stain). **(b)** Photomicrograph shows the incipient necrotic core with foam cell macrophages (top right) that are degenerated with the release of free cholesterol from cell membranes forming crystals (bottom left). **(c)** Photomicrograph shows a fully developed necrotic core *(NC),* with thinning of the fibrous cap (arrows). **(d)** Photomicrograph shows an occlusive thrombus *(Thr)* within the arterial lumen, with a disrupted fibrous cap (arrow) overlying the necrotic core *(NC)*. **(e)** Photomicrograph shows acute MI, transmural, with rupture. The rupture track is toward the left. The dark blue areas are degenerative neutrophils. **(f)** Photomicrograph shows a higher magnification of the blue areas in **e** that are fragmenting neutrophils. **(g)** Photomicrograph shows a healing infarct, with fibroblasts, early collagen strands, and abundant hemosiderin macrophages. **(h)** Photomicrograph shows hemosiderin macrophages (arrows) that were depicted at a lower resolution in **g**. **(i)** Photomicrograph shows a healed transmural infarct. This Masson trichrome stain demonstrates dense scar (blue area) from the endocardium (above) to the epicardium (below). There is some viable entrapped cardiac muscle near the endocardium (dark red area). Much of the fat near the epicardium is secondary to the infarct; fatty metaplasia is common in cardiac scars.

Endothelial activation results in adhesion and absorption of lipid within the intima of the arterial wall, which initiates a cascade eventually leading to atheroma formation. The lipid within the plaque includes two types: *(a)* esterified lipoproteins that accumulate through absorption of low-density lipoproteins (LDLs) from the blood and *(b)* nonesterified lipids and cholesterol that develop later on. Lipoproteins attract and are engulfed by macrophages that transform into foam cells filled with lipid droplets (Fig 1a). Nonesterified or free cholesterol is largely the result of the cellular breakdown of smooth muscle cells, red blood cells, and inflammatory cells within the developing core, whose cell membranes are rich in cholesterol. Histologically, cholesterol accumulates as enlarging crystals within the atherosclerotic core (Fig 1b). As the inflammatory process continues, the lesion grows and there is subsequent remodeling of the arterial wall owing to elaboration of matrix metalloproteinases. In addition, cytokines stimulate angiogenesis within the plaque and adventitia, promoting plaque growth and intraplaque hemorrhage (13). As the necrotic core enlarges, the surface of the plaque may become smaller, resulting in thinning of the fibrous cap (Fig 1c) (11,12,14).

Progressive nonocclusive thrombi, many caused by small fibrous cap ruptures, cause progressive narrowing of the arterial lumen (15). Surface endothelial erosions in the absence of plaque rupture, presumably resulting from spasm, also result in thrombi that promote the proliferation of smooth muscle cells and increase in plaque size. In some cases, especially in segments with significant narrowing by underlying plaque, the thrombus is occlusive or nearly occlusive, resulting in an MI (Fig 1d). The enlargement of the plaque is therefore the result of a variety of stimuli, including successive thrombi, intraplaque hemorrhage, and smooth muscle cell proliferation (16). Dystrophic calcification secondary to inflammation and cellular breakdown initially occurs in apoptotic smooth muscle cells in the necrotic core of fibrous plaques and can become a dominant element in the plaque (12,14). Chronic total occlusions are coronary arterial segments that are permanently blocked without significant blood flow and may be composed of dense calcified plaque or softer organized thrombus.

Ischemia results when there is insufficient oxygenated blood flow to the working myocardium and can be caused by decreased oxygen within the blood or decreased flow of blood. Reversible ischemia occurs when there is a temporary increase in oxygen demand, for example during exercise or vasodilator stress testing. Reversible ischemia clinically manifests as angina pectoris and has no histologic correlates. Irreversible ischemia indicates myocyte necrosis and cell death, which causes the release of myocyte proteins within the blood. Foci of irreversible ischemia may be microscopic, for example in unstable angina, or involve larger areas of myocardium, resulting in prolonged electrocardiographic changes and the formation of persistent ST-segment elevations on an electrocardiogram. Myocardial infarcts progress from acute histologic changes of myocyte necrosis with an acute inflammatory infiltrate (Fig 1e, 1f) to a healing phase, characterized by chronic inflammation, capillary ingrowth, and early collagen deposition (Fig 1g, 1h), and finally to a healed phase, characterized by dense scar (Fig 1i). Acute infarcts last up to 3 days, healing infarcts last from 3 days to 3 months, and healed infarcts are seen at 3 months. There are no histologic findings in infarcts that are less than 12–24 hours in duration (12,14,17).

As detailed in the following sections, CT is exquisitely sensitive for coronary artery calcium, can help detect “soft plaque” corresponding to noncalcified portions of atheroma, and can image coronary artery stenoses well enough to manage CAD and detect arterial wall remodeling. MRI can help characterize some subcomponents of atheroma in large vessels like the carotid arteries but is less appliable to coronary atheroma and is not currently recommended for clinical coronary artery stenosis imaging. All noninvasive cardiac stress perfusion imaging methods can aid in the detection of perfusion defects associated with either MI or stress-induced perfusion defects associated with physiologically significant coronary stenosis. While SPECT MPI, PET MPI, and to a lesser degree CT can detect MI, MRI has emerged as the best clinical method available. CT and MRI can also help detect fatty metaplasia associated with MI.

## Noninvasive Anatomic Assessment of Coronary Artery Stenosis

**Figure 2.**
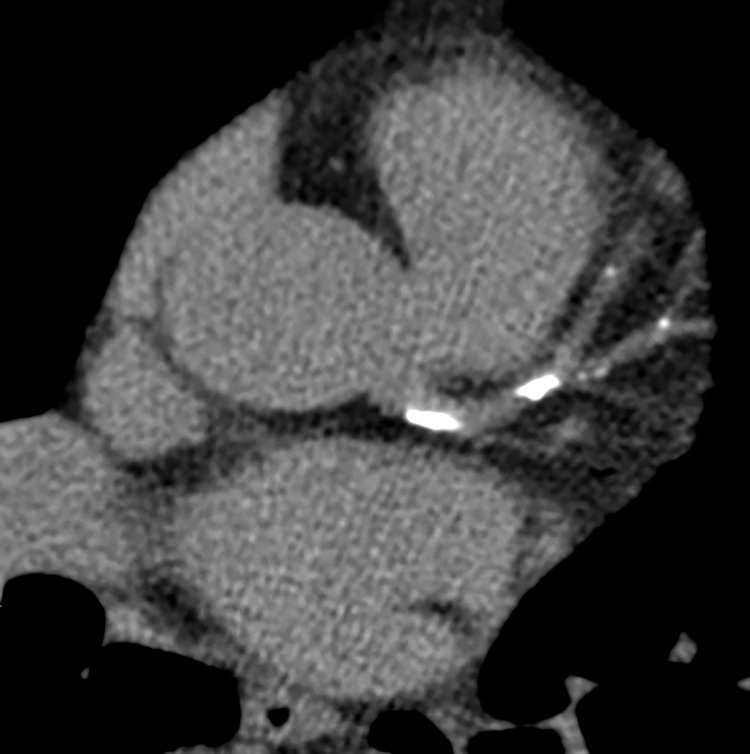
Coronary calcium scoring in a 64-year-old woman with diabetes mellitus and intermittent chest pressure. Selected axial CT image from a coronary calcium score examination shows calcification within the left main coronary artery, left anterior descending (LAD) coronary artery, and diagonal branches. The total quantified coronary calcium was severe, yielding an Agatston score of 622, which represents the 95th percentile for patients of the same age, gender, and race/ethnicity who are free of clinical cardiovascular disease and treated diabetes per the MESA coronary calcium calculator *(https://www.mesa-nhlbi.org/Calcium/input.aspx).*

CCTA is the main noninvasive imaging test used to anatomically detect coronary atherosclerotic plaque. The CCTA examination has two parts: *(a)* obtaining the coronary calcium score and *(b)* performing CCTA. The coronary calcium score is obtained with a noncontrast electrocardiographically (ECG)-gated CT examination of the heart that quantifies the amount of coronary calcium. The quantified coronary calcium score, known as the Agatston score, can be used in conjunction with reference databases that have stratified Agatston scores by age, gender, and race/ethnicity (eg, Multi-Ethnic Study of Atherosclerosis [MESA], *https://www.mesa-nhlbi.org/Calcium/input.aspx*) to risk stratify patients on the basis of the likelihood of having a coronary event within the next 5 years (Fig 2) (18,19).

After the coronary calcium score examination, an ECG-gated CCTA examination of the heart is performed during the administration of intravenous iodinated contrast material at a rate of 5–7 mL/sec. CCTA can be performed prospectively (x-ray only turned on during part of the cardiac cycle, usually mid diastole) in patients with a regular heart rate less than 60 beats per minute. Retrospective acquisition (x-ray turned on during the entire cardiac cycle) is typically used for patients with arrhythmias or higher heart rates, but it increases radiation exposure to the patient. Detailed guidelines for CCTA patient preparation and CT scanner requirements are available from the Society of Cardiovascular Computed Tomography (20).

CCTA directly aids in the visualization of coronary artery plaque, which may be noncalcified, partially calcified, or completely calcified. Noncalcified plaque is also referred to as soft plaque. Soft lipid-rich plaque tends to have lower Hounsfield units than soft predominately fibrous plaque (11–99 HU vs 77–121 HU) (21). Low-density plaque that is less than 30 HU has been described as vulnerable plaque that has a high risk of rupture. Additional vulnerable plaque features include spotty calcification, presence of positive remodeling, and/or a “napkin ring” sign (22). Positive remodeling refers to the compensatory enlargement of a coronary artery in relation to the plaque area, such that the diameter of the coronary lumen plus the plaque is greater in size than that of normal vessel segments proximal or distal to the plaque. A napkin ring sign refers to plaque that contains a central area of lower attenuation surrounded by a thin rim of high attenuation (18,22,23).

**Figure 3.**
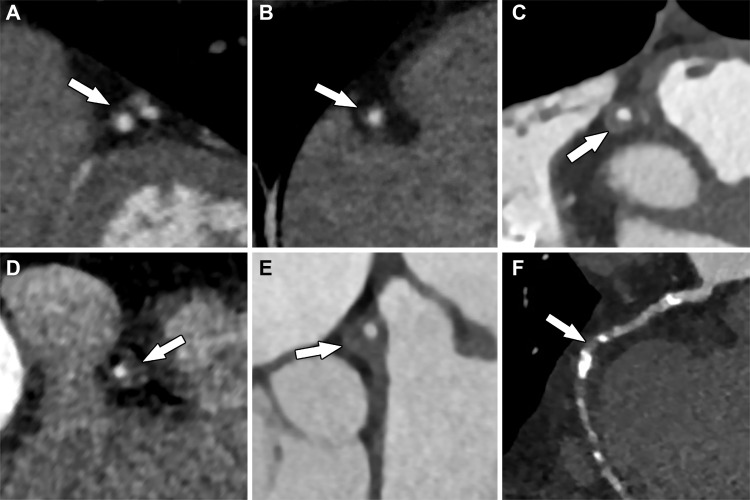
Multiplanar reformatted images of the coronary arteries from CCTA show various degrees of stenosis (arrows): *A,* approximately 10% stenosis of the left anterior descending coronary artery, consistent with minimal coronary stenosis; *B,* approximately 25% stenosis of the right coronary artery, consistent with mild coronary stenosis; *C,* approximately 55% stenosis, as well as positive remodeling of the right coronary artery, consistent with moderate stenosis; *D,* approximately 75% stenosis of the right coronary artery, consistent with severe stenosis; *E,* approximately 65% stenosis and positive remodeling of the left main coronary artery, consistent with severe stenosis; and, *F,* completely occluded mid right coronary artery that is supplied distally by collateral vessels.

The degree of luminal diameter narrowing (expressed as a percentage) resulting from the coronary artery plaque detected at CCTA determines the stenosis grade: no stenosis (0%), minimal (1%–24%), mild (25%–49%), moderate (50%–69%), severe (70%–99%), or occluded (100%) (Fig 3). In 2016, the Coronary Artery Disease Reporting and Data System (CAD-RADS) was created to standardize grading and reporting of coronary artery stenosis. CAD-RADS categories range from 0 to 5 and are based on the grade of coronary stenosis (22). Retrospectively gated CCTA can also assess left ventricular (LV) wall motion abnormalities and systolic function.

CCTA has excellent spatial resolution and can anatomically detect coronary artery plaque with high sensitivity (94%) and specificity (83%). When directly compared with invasive angiography, CCTA has an accuracy of 95% for detecting coronary artery stenoses. However, the greatest strength of CCTA lies in its excellent negative predictive value (99%), established by the multicenter ACCURACY (Assessment by Coronary Computed Tomographic Angiography of Individuals Undergoing Invasive Coronary Angiography) trial (24). Subsequently, multiple large clinical trials, including ACRIN-PA (American College of Radiology Imaging Network PA), ROMICAT II (Rule Out Myocardial Ischemia/Infarction by Computer Assisted Tomography), and CT-STAT (Coronary Computed Tomographic Angiography for Systematic Triage of Acute Chest Pain Patients to Treatment), have analyzed patients with chest pain in the emergency department and found that a negative CCTA in low- to intermediate-risk patients safely excluded CAD (25–27). In addition, in a patient with a negative CCTA examination, the 2-year and 3-year major adverse cardiovascular events (MACE) risk has been reported as 0% and 0.8%, respectively (28,29). Although CCTA has high sensitivity and specificity for detection of CAD, it only provides an anatomic assessment, and thus its positive predictive value remains poor (48%–64%), often necessitating further functional assessment of the detected CAD (24).

**Figure 4.**
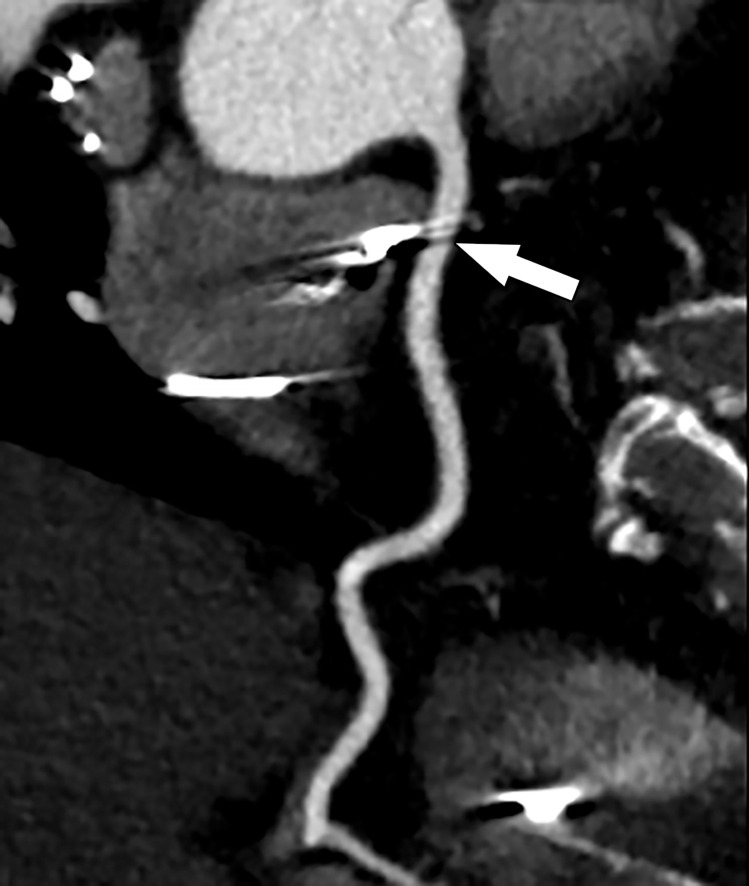
Curved multiplanar reformatted image from CCTA shows beam-hardening artifact (arrow) within the right coronary artery, secondary to a pacemaker lead within the right atrium.

A few notable common challenges of CCTA include cardiac motion artifacts related to high heart rate (>65 beats per minute) and/or arrhythmia, calcium blooming artifacts (calcium appears falsely larger, leading to overestimation of stenosis severity), and beam-hardening artifact (dark bands adjacent to high-attenuation areas such as metal or dense contrast material) (Fig 4). Problem-solving strategies include *(a)* administering oral and/or intravenous β-blockers to lower heart rate before imaging, *(b)* removing data collected on ectopic beats through ECG editing software after imaging, *(c)* performing retrospective imaging for significant arrhythmia noted before the examination, *(d)* using iterative reconstruction algorithms to reduce calcium blooming, and *(e)* using a saline bolus chaser (20,30,31). Recently, some vendors have begun developing metal artifact reduction postprocessing software, and preliminary data are promising (32).

MR angiography (MRA) has been suggested as a noninvasive method to detect CAD. MRA involves no ionizing radiation, and the lumen of the artery is well depicted on MRA images, even in the presence of heavy calcification. MRA has a sensitivity and specificity of 93% and 42%, respectively, for the detection of coronary disease (33). Current free-breathing MRA protocols that obtain submillimeter isotropic resolution suffer from long imaging times, which makes it a less feasible examination (33,34). However, more recently, there has been an increased amount of research to improve the speed of acquisition times (parallel imaging, compressed sensing, translational respiratory motion correction with two-dimensional and three-dimensional image-based navigators, and undersampled two-dimensional and three-dimensional patch-based reconstruction algorithms). Preliminary results for these techniques are promising, but these require continued research and validation (34–37).

## Noninvasive Functional Assessment of Coronary Artery Stenosis

Over the past few years, there has been significant advancement in noninvasive imaging methods that now allow the accurate noninvasive functional assessment of CAD. These methods include stress perfusion imaging, dobutamine stress cardiac MRI, and stress echocardiography. Although not an imaging method, an exercise ECG test is a common initial noninvasive test performed for risk stratification that deserves brief mention. This test has the patient exercise on a treadmill while attached to an ECG machine and looks for ECG changes suggestive of ischemia while the patient is exercising. Exercise ECG has lower sensitivity (50%–68%) but moderate to good specificity (77%–90%) (38).

Of the noninvasive imaging methods, stress perfusion imaging methods (MRI, CT, PET, and SPECT) have become the most widely used. Some of these modalities can also help assess myocardial viability to varying degrees, dependent on the examination protocols. Indications and contraindications for stress perfusion imaging are listed in Table 1 (39,40). In general, two sets of myocardial perfusion images of the heart are obtained during stress perfusion imaging: stress and rest. Stress images are obtained during the administration of a vasodilator medication (or at peak exercise stress), coordinated with an injection of contrast or radionuclide material. Commonly used vasodilator medications and protocols include the following (39):

1. Adenosine (140 µg/kg/min) intravenous infusion for up to 6 minutes, although higher-dose protocols have been proposed to manage inadequate response to the standard dose. Adenosine has a short half-life and usually does not require reversal agents.

2. Regadenoson (0.4 mg/5 mL) intravenous injection followed by saline (5 mL) intravenous injection. Regadenoson can be reversed after the stress procedure is completed with an intravenous injection of 100 mg of aminophylline.

3. Dipyridamole (0.56 mg/kg) intravenous infusion over 4 minutes (up to a maximum of 60 mg). Dipyridamole has a much longer half-life than aminophylline, so there are reports of patients having recurrent symptoms or adverse events after leaving an imaging center despite initial reversal with aminophylline.

**Table 1:**
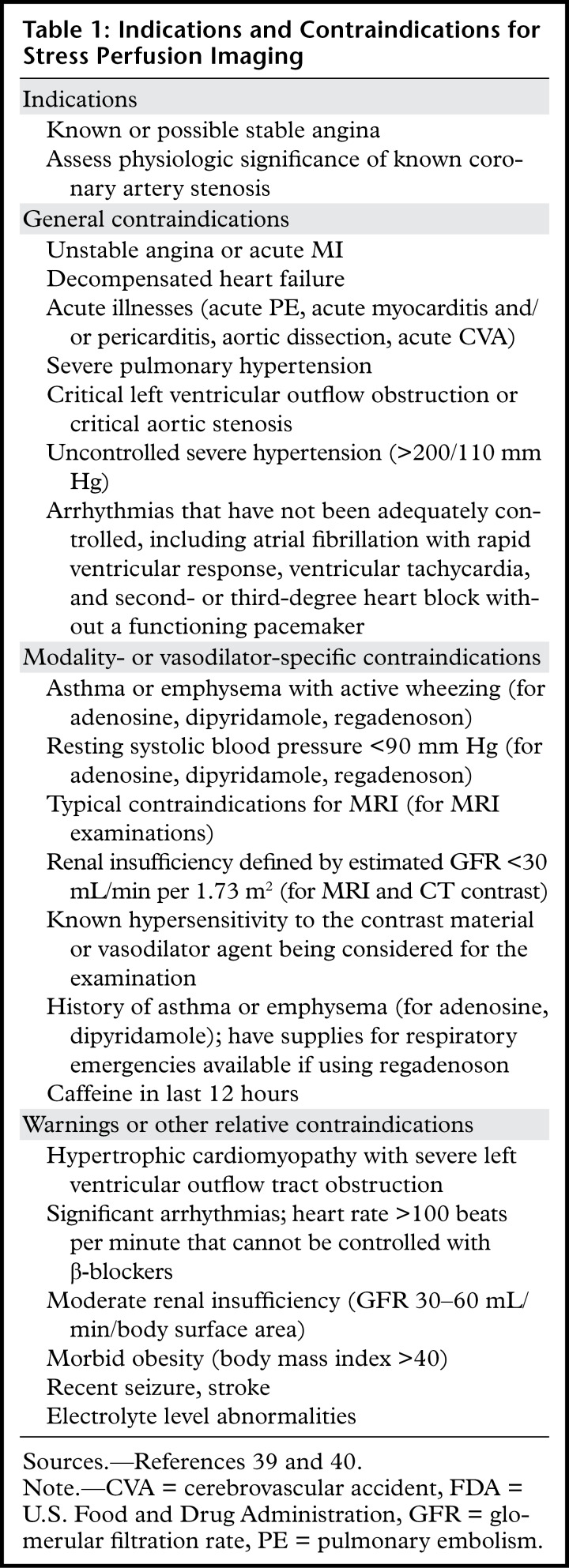
Indications and Contraindications for Stress Perfusion Imaging

Rest perfusion images are obtained when the heart is at rest during administration of contrast or radionuclide material. Rest images may be obtained either before the stress images or after the stress images once the heart rate has returned to baseline.

Vasodilators have a good safety profile but can cause significant adverse events in a small fraction of patients and are contraindicated in some conditions. Adenosine can cause second- and third-degree atrioventricular block in 8% of cases and can also precipitate bronchospasm. Thus, adenosine is contraindicated in patients with second-degree atrioventricular block and in patients with severe asthma or emphysema. Regadenoson is a selective alpha-2A (A_2A_) adenosine receptor agonist with coronary vasodilator properties and thus avoids heart block and is not contraindicated in patients with asthma and emphysema, but these patients need to be monitored carefully, with emergency treatments for bronchospasm available. Dipyridamole vasodilates primarily by raising platelet cyclic adenosine monophosphate (cAMP) levels via inhibition of its breakdown by cyclic nucleotide phosphodiesterase and also by blocking uptake of adenosine and thus has similar contraindications as those for adenosine (39).

### Cardiac MR Stress Perfusion Imaging

Cardiac MR stress perfusion imaging is the most widely used MRI stress imaging technique for the heart. Conventional cardiac MR stress perfusion examinations image the wash-in and washout of gadolinium contrast material within the myocardium during administration of a vasodilator agent (stress), as well as at rest. Multiple prior studies have shown that cardiac MR stress perfusion imaging has excellent diagnostic accuracy for CAD (41–43). Either stress or rest perfusion images may be obtained first. Typically at the authors’ institution, conventional MR stress perfusion imaging uses a saturation recovery pulse with fast low-angle shot (FLASH) read out and obtains three short-axis images (basal, mid, and apical) during the administration of a vasodilator agent (adenosine, regadenoson, or dipyridamole) and administration of gadolinium contrast material (0.05 mmol/kg diluted with saline to 10-mL volume) at 2 mL/sec during a 60 cardiac cycle acquisition time. This yields a total of 60 images at each location (180 images total). The timing of the intravenous gadolinium contrast material administered during the vasodilator stress procedure depends on the vasodilator agent used, and it is administered ~3 minutes after the adenosine infusion started, ~70 seconds after regadenoson injection, or 4 minutes after dipyridamole. It should be noted that gadolinium may be injected at a rate of up to 5 mL/sec for perfusion imaging, but decreasing the flow rate to 2 mL/sec has an advantage if quantitative perfusion is subsequently desired (described further in this article). Rest perfusion images are obtained at the same LV short-axis locations approximately 10 minutes after stress images and during administration of gadolinium contrast material (0.05 mmol/kg diluted with saline to 10-mL volume) at 2 mL/sec during a 60 cardiac cycle acquisition time, again yielding 60 images per location (180 images total).

Cine MR images are obtained between stress and rest perfusion acquisitions at the authors’ institution. A third optional gadolinium dose of 0.05 mmol/kg (diluted with saline to 10 mL), referred to as a top-off dose, is commonly administered after the rest perfusion images in preparation for late gadolinium enhancement (LGE) images, which are obtained approximately 10 minutes after rest perfusion images. Administration of the optional top-off dose of gadolinium yields a total dose of 0.15 mmol/kg of gadolinium for the entire conventional cardiac MR stress perfusion examination. If the optional top-off dose is not administered, LGE images should be obtained earlier, approximately 5 minutes after the rest perfusion images. LGE images obtained as part of the cardiac MRI stress test can help detect clinically unrecognized MI, which is also a sign of physiologically significant CAD (9).

The perfusion sequence described previously (saturation recovery pulse with FLASH read out) is commonly used. However, there are other options as well. Technical considerations for FLASH, steady-state free-precession (SSFP), and hybrid gradient-echo echo-planar perfusion methods have been reviewed previously (44) and are beyond the scope of this article. For comparable spatial resolution, temporal resolution, and spatial coverage achievable during typical stress perfusion heart rates (100–120 beats per minute), some generalizations become clear. First, the short cardiac cycle during stress perfusion makes it difficult to image more than three locations per heartbeat. SSFP imaging tends to have the highest contrast-to-noise ratio (CNR), and FLASH imaging has the lowest CNR. Saturation pulses must be carefully selected, particularly for quantitative analyses. Finally, imaging parameters can strongly influence image artifacts, particularly in the LV cavity, which is important for quantification but also can introduce artifacts that adversely affect the myocardium.

Conventional cardiac MR stress perfusion examinations are generally reported by using the 17-segment American Heart Association model, with the apex excluded (45). Qualitative visual assessment examines the short-axis perfusion images obtained at stress and rest for areas of low signal intensity or slower enhancement during the first 7–15 heartbeats after arrival of contrast material and that fall within the distribution of a coronary artery (Fig 5), but a qualitative assessment may miss balanced ischemia. The description of the perfusion defect should comment on the severity of the defect (mild, moderate, or severe), transmural extent (subendocardial or transmural), and if it is a reversible defect. A reversible defect is one that is depicted on stress images but not on rest images. Care must be taken not to mistake dark rim artifacts for true perfusion defects. Dark rim artifacts (Fig 6) are subendocardial areas of low signal intensity that appear on stress images, commonly in the interventricular septum. They are much thinner than true subendocardial perfusion defects and have various causes, including Gibbs ringing, motion, partial volume errors, and nonuniformity of k-space. High heart rates tend to make dark rim artifacts worse (46).

**Figure 5a.**
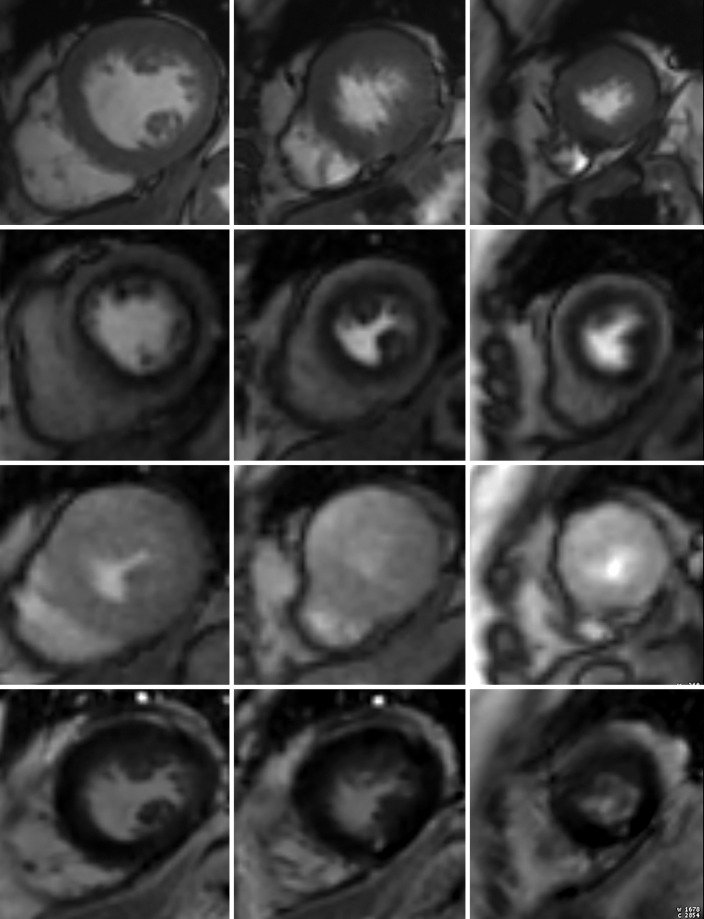
Stress perfusion imaging in a 53-year-old woman with chest pain on exertion and at rest. **(a)** From left to right in the top row, basal, mid, and apical short-axis cine MR images of the LV show normal global and regional left ventricular systolic function (LV ejection fraction, 61%). In the second row, adenosine stress perfusion images of the same LV locations as in the top row show severe subendocardial perfusion defects involving all myocardial segments. In the third row, the rest perfusion images of the same LV locations are normal. In the fourth row, the LGE images of the same short-axis LV locations show a small focus of enhancement that may represent a microinfarction or an embolic infarction in the mid to apical inferior wall. **(b, c)** Left anterior oblique (LAO) **(b)** and LAO caudal **(c)** invasive coronary angiographic images show severe proximal and distal right coronary artery stenoses on the LAO view (arrows in **b**), as well as severe stenosis of the distal left main coronary artery on the LAO caudal view (arrow in **c**).

**Figure 5b.**
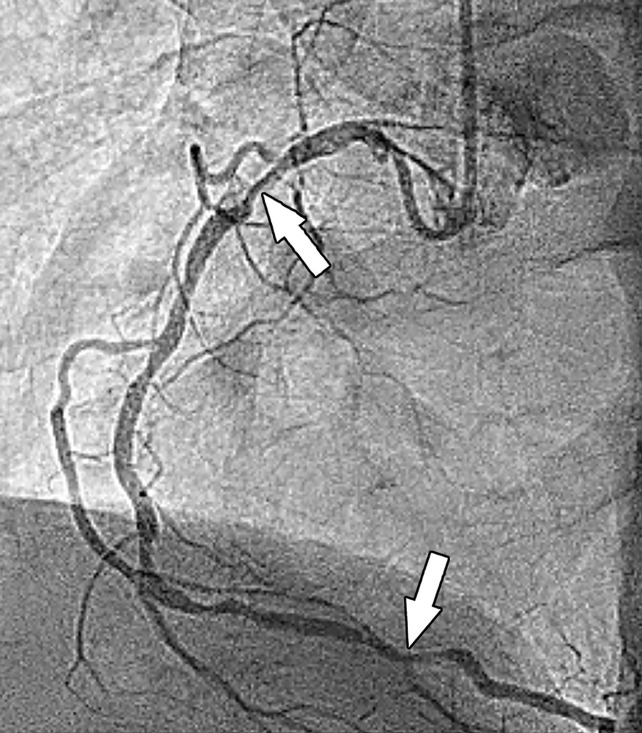
Stress perfusion imaging in a 53-year-old woman with chest pain on exertion and at rest. **(a)** From left to right in the top row, basal, mid, and apical short-axis cine MR images of the LV show normal global and regional left ventricular systolic function (LV ejection fraction, 61%). In the second row, adenosine stress perfusion images of the same LV locations as in the top row show severe subendocardial perfusion defects involving all myocardial segments. In the third row, the rest perfusion images of the same LV locations are normal. In the fourth row, the LGE images of the same short-axis LV locations show a small focus of enhancement that may represent a microinfarction or an embolic infarction in the mid to apical inferior wall. **(b, c)** Left anterior oblique (LAO) **(b)** and LAO caudal **(c)** invasive coronary angiographic images show severe proximal and distal right coronary artery stenoses on the LAO view (arrows in **b**), as well as severe stenosis of the distal left main coronary artery on the LAO caudal view (arrow in **c**).

**Figure 5c.**
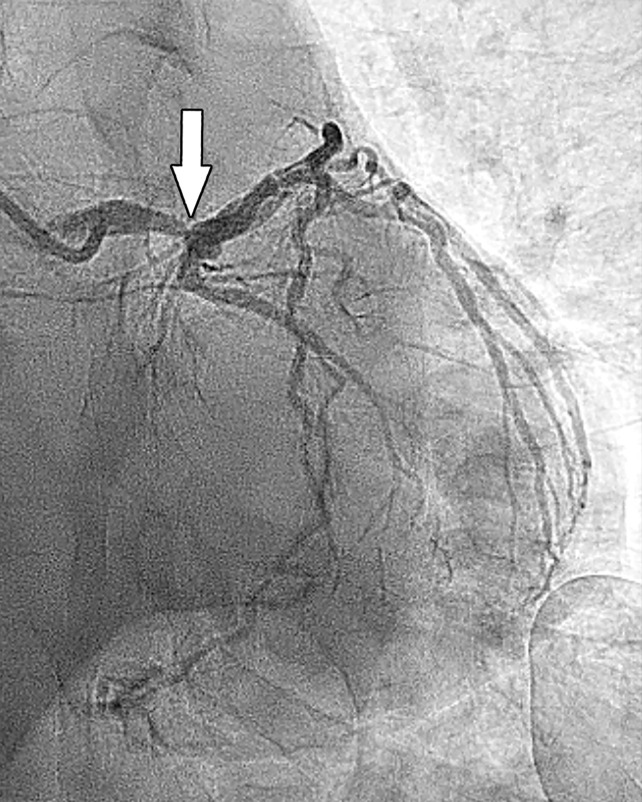
Stress perfusion imaging in a 53-year-old woman with chest pain on exertion and at rest. **(a)** From left to right in the top row, basal, mid, and apical short-axis cine MR images of the LV show normal global and regional left ventricular systolic function (LV ejection fraction, 61%). In the second row, adenosine stress perfusion images of the same LV locations as in the top row show severe subendocardial perfusion defects involving all myocardial segments. In the third row, the rest perfusion images of the same LV locations are normal. In the fourth row, the LGE images of the same short-axis LV locations show a small focus of enhancement that may represent a microinfarction or an embolic infarction in the mid to apical inferior wall. **(b, c)** Left anterior oblique (LAO) **(b)** and LAO caudal **(c)** invasive coronary angiographic images show severe proximal and distal right coronary artery stenoses on the LAO view (arrows in **b**), as well as severe stenosis of the distal left main coronary artery on the LAO caudal view (arrow in **c**).

**Figure 6.**
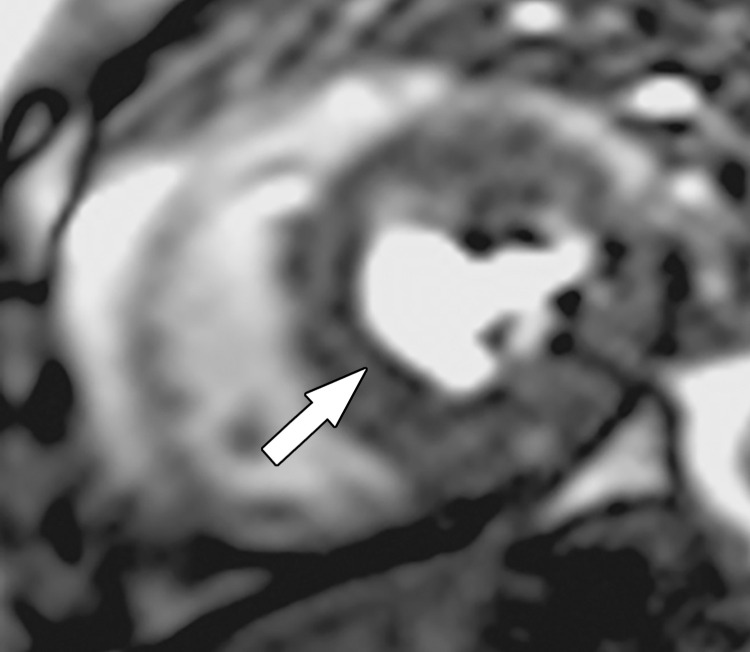
Dark rim artifact at stress perfusion imaging in a 48-year-old woman with a history of Poland syndrome and atypical chest pain. Mid left ventricular short-axis stress perfusion image shows a thin subendocardial area of low signal intensity (arrow) in this patient with normal coronary arteries, consistent with a dark rim artifact.

Semiquantitative methods include the contrast enhancement ratio (CER = [peak signal intensity of myocardial segment − baseline signal intensity of myocardial segment]/baseline signal intensity of myocardial segment), myocardial to LV upslope ratio (initial upslope of myocardial time-intensity curve of the segment of interest divided by the initial upslope of the LV time-intensity curve), and upslope integral (area under the curve for a baseline adjusted myocardial time–signal intensity curve). Currently, semiquantitative methods are not recommended because they underestimate myocardial perfusion at high blood flow rates and do not significantly improve diagnostic accuracy when compared with qualitative assessment (47–49).

More recently, fully quantitative MR stress perfusion methods, which outperform both qualitative and semiquantitative assessments, have been extensively validated by microspheres and coronary angiography (47,48,50). Fully quantitative methods have the highest diagnostic accuracy of all cardiac MRI stress perfusion analysis methods, are accurate across a wide range of myocardial blood flow rates, and quantify myocardial blood flow in milliliters per minute per gram of tissue at the pixel level (47,48). Quantitative perfusion maps are generated in-line on the MR imager or automatically through commercial postprocessing software (28–30). The perfusion quantification is based on the indicator dilution theory applied to cardiac MRI first-pass perfusion (50–54).

To perform fully quantitative perfusion imaging, both the arterial input function and the transit of contrast material through the myocardium must be accurately measured. The arterial input function is the delivery of contrast material to the heart, as visualized in LV blood pool cavity. This can be performed by two different methods: dual-bolus method and dual sequence method.

***Dual-Bolus Method.—***The dual-bolus method uses the standard perfusion sequence but two different boluses of contrast material: a lower-concentration bolus optimized for the arterial input function (10-fold dilution, net dose 0.005 mmol/kg) and a higher-concentration bolus optimized for the myocardium (not diluted, net dose 0.05 mmol/kg). Both doses of contrast material are flushed with saline into equal volumes injected at the same rate (typically 2–4 mL/min). Thus, both stress and rest perfusion examinations are run twice (once for each bolus), yielding a total of four sets of perfusion images (lower-concentration bolus stress, higher-concentration bolus stress, lower- concentration bolus rest, and higher-concentration bolus rest) (50). Proton density–weighted images are obtained at the beginning of the perfusion sequence to normalize signal intensity values and to correct surface coil intensity variation.

***Dual-Sequence Method.—***The dual-sequence method uses two different sets of image acquisition parameters in a saturation recovery perfusion sequence, a low spatial resolution sequence optimized for the arterial input function with FLASH readout and a high-resolution sequence optimized for the myocardium with SSFP or FLASH readout) and only one dose of contrast material. For each cardiac cycle, one low-resolution LV short-axis image for the arterial input function and three high-resolution LV short-axis perfusion images of the myocardium (base, mid, and apical) are obtained (52). Proton density–weighted images are obtained at the beginning of the perfusion sequence to normalize signal values and surface coil intensity correction.

Once the arterial input function and the myocardial perfusion images are obtained, by either the dual-bolus or dual-sequence method, a motion correction algorithm is applied to the images. Artificial intelligence automatically segments the LV cavity and myocardium, detects both the arterial input function and myocardium, and extracts time-intensity curves. Fermi function constrained deconvolution is applied, which converts the time-intensity curve data into myocardial blood flow pixel maps that depict myocardial blood flow in milliliters per minute per gram of tissue on a per pixel basis. Additionally, polar maps depicting the stress myocardial blood flow, rest myocardial blood flow, and myocardial perfusion reserve (MPR) are also generated (Fig 7) (48,51). MPR is the ratio of the stress myocardial blood flow to the rest myocardial blood flow. Absolute myocardial blood flow by fully quantitative cardiac MR stress perfusion imaging can differentiate transmural defects, subendocardial defects, and normal transmural flow. Similar to PET, MPR less than 1.5 is abnormal, 1.5–2 is borderline, and greater than 2 is normal (47,51). In the absence of epicardial CAD, fully quantitative cardiac MR perfusion imaging can also aid in the identification of microvascular ischemia (55). Fully quantitative perfusion methods can also differentiate dark rim artifacts from true perfusion defects (46).

**Figure 7a.**
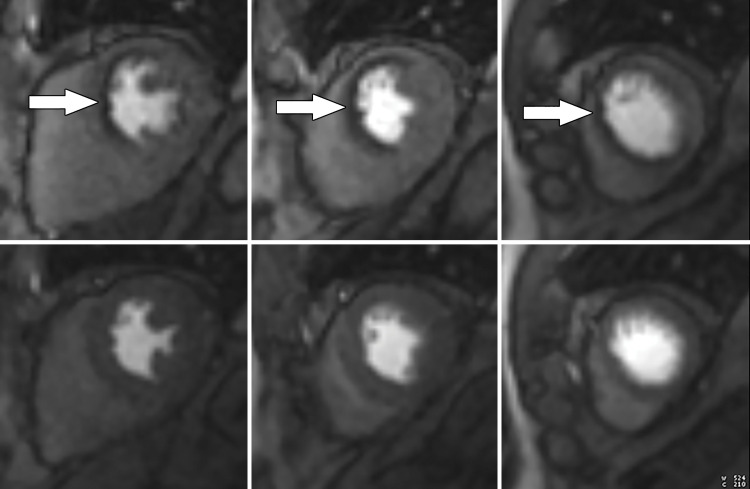
Abnormal ECG results in a 65-year-old man. **(a)** Short-axis adenosine stress perfusion cardiac MR images (top row) show a severe subendocardial perfusion defect in the LAD territory that becomes more transmural toward the apical segments (arrows). The rest perfusion images (bottom row) are normal. **(b)** Quantitative perfusion software automatically generates pixel maps that show myocardial perfusion in milliliters per gram per minute at stress (top row) and rest (bottom row) in this patient with an LAD coronary artery occlusion. Note that within the LAD territory, there is no significant change in perfusion (arrows) on the stress images when compared with the rest images, as both are less than about 1 mL/min/g (blue-green color range). This corresponds to the perfusion defect depicted on the raw MRI perfusion images. The normal myocardium reaches the orange color range during stress. **(c)** Polar maps, also automatically generated from the myocardial perfusion pixel maps, show myocardial blood flow during stress (left) and at rest (middle). The map on the right shows the myocardial perfusion reserve *(MPR)*. All LAD segments have an abnormal MPR (abnormal =MPR <1.5), corresponding to the patient’s LAD coronary artery occlusion.

**Figure 7b.**
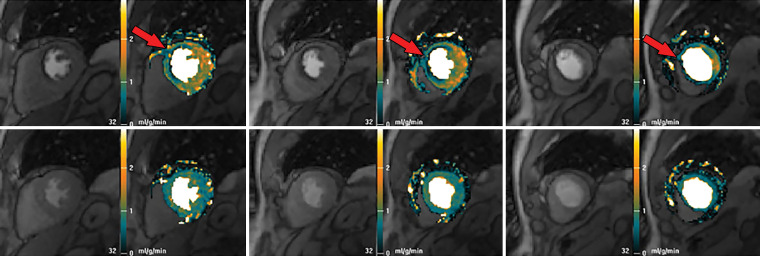
Abnormal ECG results in a 65-year-old man. **(a)** Short-axis adenosine stress perfusion cardiac MR images (top row) show a severe subendocardial perfusion defect in the LAD territory that becomes more transmural toward the apical segments (arrows). The rest perfusion images (bottom row) are normal. **(b)** Quantitative perfusion software automatically generates pixel maps that show myocardial perfusion in milliliters per gram per minute at stress (top row) and rest (bottom row) in this patient with an LAD coronary artery occlusion. Note that within the LAD territory, there is no significant change in perfusion (arrows) on the stress images when compared with the rest images, as both are less than about 1 mL/min/g (blue-green color range). This corresponds to the perfusion defect depicted on the raw MRI perfusion images. The normal myocardium reaches the orange color range during stress. **(c)** Polar maps, also automatically generated from the myocardial perfusion pixel maps, show myocardial blood flow during stress (left) and at rest (middle). The map on the right shows the myocardial perfusion reserve *(MPR)*. All LAD segments have an abnormal MPR (abnormal =MPR <1.5), corresponding to the patient’s LAD coronary artery occlusion.

**Figure 7c.**
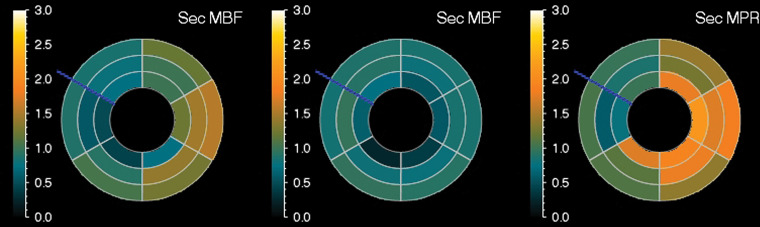
Abnormal ECG results in a 65-year-old man. **(a)** Short-axis adenosine stress perfusion cardiac MR images (top row) show a severe subendocardial perfusion defect in the LAD territory that becomes more transmural toward the apical segments (arrows). The rest perfusion images (bottom row) are normal. **(b)** Quantitative perfusion software automatically generates pixel maps that show myocardial perfusion in milliliters per gram per minute at stress (top row) and rest (bottom row) in this patient with an LAD coronary artery occlusion. Note that within the LAD territory, there is no significant change in perfusion (arrows) on the stress images when compared with the rest images, as both are less than about 1 mL/min/g (blue-green color range). This corresponds to the perfusion defect depicted on the raw MRI perfusion images. The normal myocardium reaches the orange color range during stress. **(c)** Polar maps, also automatically generated from the myocardial perfusion pixel maps, show myocardial blood flow during stress (left) and at rest (middle). The map on the right shows the myocardial perfusion reserve *(MPR)*. All LAD segments have an abnormal MPR (abnormal =MPR <1.5), corresponding to the patient’s LAD coronary artery occlusion.

Cardiac MR stress perfusion imaging offers many advantages but has some disadvantages. It boasts high sensitivity and specificity of 90% and 94%, respectively (56). There is no radiation involved, and cardiac MR stress perfusion imaging can diagnose a wide variety of cardiac diseases in addition to evaluating for functionally significant CAD. Additional sequences obtained during the examination (cine MRI and LGE) provide information on wall motion abnormalities, systolic function, and areas of fibrosis. Disadvantages include a time-consuming workflow that may make it difficult to implement in some centers with limited staffing available for this test. Additionally, some implanted devices may preclude cardiac MRI, and administration of gadolinium contrast material is not desirable in patients with severe renal failure. Cardiac MR stress perfusion imaging is growing in popularity. However, its availability is not yet ubiquitous.

Although not nearly as common as cardiac MR stress perfusion imaging, dobutamine stress cardiac MRI tests should be mentioned. Dobutamine, a synthetic β-adrenergic agent, is infused starting at a dose of 5 μg/kg/min and increased incrementally every 3 minutes to 10, 20, 30, and 40 μg/kg/min and stopped as soon as 85% or more of age-predicted heart rate is achieved. Atropine (0.25-mg doses to a maximum of 1 mg) can be given as a supplement to help boost heart rate response. Cine MR images are obtained at peak stress, and development or worsening of a wall motion abnormality (hypokinesis, akinesis, or dyskinesis) when compared with the rest images is considered a positive test result for functionally significant CAD. Dobutamine stress cardiac MRI has good performance (sensitivity 86%–89%, specificity 80%–86%). A major advantage of this technique is that it does not involve radiation exposure or gadolinium contrast material; thus it can be performed in patients with end-stage renal disease. However, it is not widely available. Dobutamine stress cardiac MRI is more likely to induce severe chest pain, ventricular tachycardia, or ventricular fibrillation than the vasodilators (39,42,57).

Additionally, there are some new techniques that are currently in development. Arterial spin labeling is a technique that quantifies myocardial blood flow without the use of contrast material. This technology uses radiofrequency pulses to alter the longitudinal magnetization of blood, thus labeling it as an endogenous tracer. This label decays at a time constant that equals the T1 relaxation time. After a delay to allow this labeled blood to affect the myocardium, images are obtained. A second set of myocardial images is obtained in the absence of the labeled blood. The two image sets are compared to quantitatively determine the amount of labeled blood flow to the myocardium (58). Contrast material–free adenosine stress and rest T1 mapping has recently been shown to distinguish among normal, ischemic, and infarcted myocardium (59). Blood-oxygen level–dependent (BOLD) imaging is a noncontrast technique that uses deoxygenated hemoglobin in the blood as contrast material along with a T2* oxygenation-sensitive sequence. BOLD images of the myocardium are obtained at rest and during the administration of a vasodilator (eg, adenosine) for stress. Decreased areas of myocardial signal intensity during stress imaging correspond to areas of ischemia (60). Further validation of these newer techniques offers promise of contrast material–free cardiac MRI stress testing someday in the future.

### CT Stress Perfusion Imaging

Improved spatial and temporal resolution of current multidetector CT systems as well as improved CT technology (wider detector coverage and new-generation iterative reconstruction) has yielded the development of modern CT stress perfusion (61). CT stress perfusion imaging is a vasodilator stress perfusion technique that combines the anatomic information obtained from CCTA and functional imaging from the CT stress perfusion into one examination that can anatomically detect and then determine the physiologic significance of CAD. CT perfusion involves obtaining two CT examinations: stress and rest. Stress CT is performed during the administration of a vasodilator agent (adenosine, regadenoson, or dipyridamole as described previously) and during the administration of intravenous iodinated contrast material. Rest CT (the CCTA) is performed during the administration of iodinated contrast material.

The CT perfusion examination has several options. Either stress or rest CT can be performed first. However, it is necessary to wait 10–20 minutes between the two parts of the examination, as each use a vasodilating agent (adenosine, regadenoson, or dipyridamole for the stress perfusion and sublingual nitroglycerin for the rest perfusion/CCTA). Both prospective and retrospective ECG gating are possible, but retrospective ECG gating is recommended for CT scanners that have a smaller z-axis (<8 cm). CT perfusion can be performed on either a single-energy or a dual-energy CT scanner. The stress CT technique can be static or dynamic (40).

***Static CT Stress Perfusion.—***A single CT image of the heart is obtained at peak myocardial perfusion during vasodilator stress, which is determined either automatically by bolus tracking or manually. Since only a single CT image is obtained, timing is critical. Comparison with the rest CT image allows qualitative assessment of perfusion defects (62). Balanced ischemia may be missed with qualitative assessment (63).

***Dynamic CT Stress Perfusion.—***Multiple CT images obtained over time and during administration of both a vasodilator agent and iodinated contrast material depict the wash-in and washout of contrast material within the myocardium. High temporal resolution (found on second- and third-generation dual-source CT scanners or later-generation single-source CT scanners) is best for this technique to be successful. For CT scanners with a shorter z-axis, shuttle mode is necessary (63). Compared with the simple static stress perfusion technique, dynamic stress perfusion tends to result in a higher radiation dose to the patient, but it is more commonly performed than static CT stress perfusion (40,64). Both semiquantitative and fully quantitative analyses are possible with dynamic CT stress perfusion. Semiquantitative analysis can be performed by the upslope method (upslope of myocardial attenuation/upslope of the blood pool attenuation) (62,65). Fully quantitative perfusion measures myocardial blood flow in milliliters per gram per minute with postprocessing software and follows the principles of the indicator dilution theory (53,54,63).

Postprocessing involves automatic (by artificial intelligence) or manual segmentation of the myocardium. Extraction of time-attenuation curves of the arterial input function (obtained from the region of interest on the descending aorta) and myocardial time-attenuation curves is performed, and myocardial blood flow is quantified by model-based deconvolution in milliliters per gram per minute per voxel (66). Both semiquantitative and fully quantitative analysis methods correlate well with myocardial blood flow, as validated by microsphere and invasive FFR experiments (65,66). However, compared with fully quantitative MR stress perfusion imaging, which has been extensively validated and is now becoming more widely adopted, fully quantitative stress CT perfusion remains an ongoing area of research (63,65,66).

Approximately 10–20 minutes after (or before, depending on if rest or stress imaging is performed first) the stress CT perfusion examination, the rest CT perfusion examination is performed. The rest perfusion CT examination is performed after the administration of sublingual nitroglycerin (for coronary artery dilation) and β-blockers (for lowering heart rate) and during the administration of intravenous iodinated contrast material. The rest CT examination obtains both the CCTA and the rest perfusion images.

The CCTA images are analyzed for plaque and/or coronary stenosis, according to the CAD-RADS scoring system (22). LV short-axis (basal, mid, and apical) and long-axis (two, three, and four chamber) CT stress and rest perfusion images are evaluated for areas of decreased perfusion on the stress perfusion images, according to the American Heart Association 17-segment model, on a postprocessing workstation (45,67). If a dual-energy CT scanner is used, iodine maps depicting areas of perfusion (or lack of perfusion) can also be produced (68).

**Figure 8.**
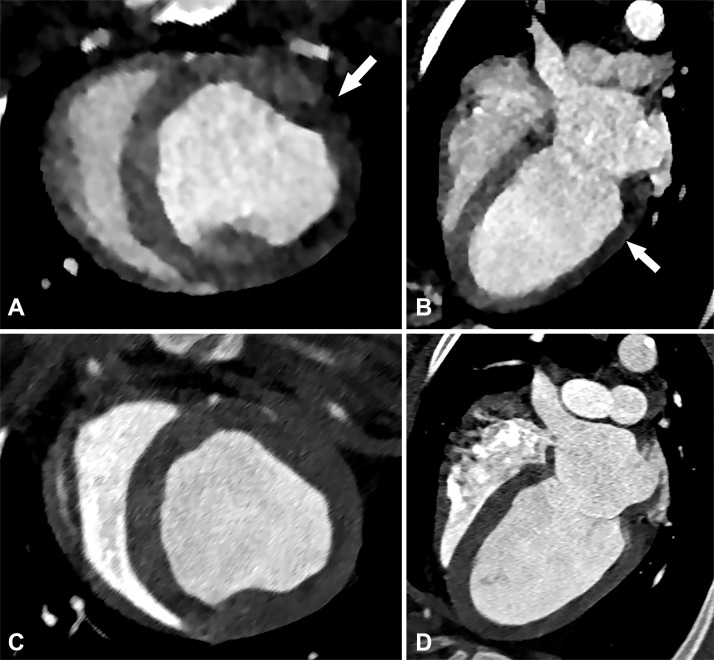
Perfusion defect in a 54-year-old man with chest pain. Short-axis, *A,* and four-chamber, *B,* CT images from a dynamic CT stress perfusion examination show a large transmural perfusion defect within the lateral wall (arrows), corresponding to a hemodynamically significant left circumflex lesion. Short-axis, *C,* and four-chamber, *D,* rest perfusion CT images are normal.

Similar to the MRI stress perfusion examination, the description of the perfusion defect should comment on the severity of the defect (mild, moderate, or severe), the size (subendocardial or transmural), and if it is a reversible defect (Fig 8). A reversible defect is one that is present on stress images but not on rest images (40). For retrospectively gated studies, LV wall motion abnormalities and function should be assessed and reported.

Artifacts should also be described. Beam- hardening artifact (dark bands in areas adjacent to metal objects or dense contrast material) is a problem depicted on both static and dynamic stress perfusion techniques. This is especially common within the basal inferolateral wall near the aorta and can be mistaken for a perfusion defect. Beam-hardening artifacts are improved with the use of dual-energy CT (69). Low-radiation-dose techniques can decrease the signal-to-noise ratio, making it difficult to discern smaller perfusion defects. The increased dose from dynamic CT stress perfusion can be somewhat reduced by using iterative reconstruction methods (63).

CT stress perfusion has good accuracy (93%) when compared with that of invasive FFR measurement. When added to CCTA, the specificity and positive predictive value of the combined examination are significantly improved (64,67,70). As CCTA has excellent negative predictive value that safely excludes CAD, the best utility of CT stress perfusion is in patients with intermediate to high pretest probability for CAD, as they would likely need additional functional assessment (64,71). Dynamic stress CT perfusion has improved sensitivity compared with that of static CT perfusion. However, its specificity and radiation dose are lower and higher, respectively (63). The major disadvantages of this technique are that it is precluded in cases of renal failure and that patients are exposed to contrast material and radiation two times.

### Fractional Flow Reserve CT

Unlike the previously described noninvasive imaging methods, FFR CT is not a cardiac stress technique but a novel postprocessing method that uses data obtained at CCTA and computational fluid dynamics to calculate FFR. This process involves the computation of coronary blood flow velocity and pressure, which both vary with time and position, from the CCTA data and requires *(a)* constructing an anatomic model of the ascending aorta and coronary artery lumen, *(b)* using a mathematical model of coronary physiology, and *(c)* using a numerical method to solve fluid dynamic equations (Navier-Stokes equations) (72,73).

The CCTA dataset provides patient-specific coronary artery anatomy (including branching patterns and pathology) used to create an anatomic model of the lumen of the coronary artery tree. The domain, or portion of the CCTA to be analyzed, is bounded by the blood flow inlet (aortic root) and the blood flow outlet (ascending aorta and coronary arteries). A segmentation algorithm analyzes the coronary arteries, identifies and segments coronary artery plaque within all vessels, and creates an anatomic model of the lumen of the entire coronary artery tree, as well as the proximal ascending aorta. High-quality CCTA data are necessary to achieve a good model of the luminal anatomy of the patient’s coronary artery tree (72).

A mathematical model of coronary physiology is necessary to determine conditions that represent microvascular resistance, aortic pressure, and coronary output. To accomplish this, lumped parameter models are used. Lumped parameter models of the heart, systemic circulation, and coronary circulation are applied to the patient-specific model from the CCTA data. The lumped model is similar to an electrical circuit and contains multiple parameters such as resistance, capacitance, inductance, etc, that are necessary to describe the relationship of blood flow and pressure. Parameter values are selected accordingly to compute cardiac output and aortic pressure (72,73). Mathematical principles governing the nonlinear relationship of vessel size and blood flow and the nonlinear inverse relationship of vessel size and blood flow resistance are used. Resistance values for each branch in the coronary tree are calculated and assigned for resting conditions (72,74). The effect that adenosine has on reducing coronary artery vessel resistance is also included (72).

Once the parameters for the coronary physiology model are defined, the anatomic model of the coronary artery tree lumen is discretized into millions of finite elements (small units of volume) for analysis (75). The solution of the Navier-Stokes equation is then computed for each element for thousands of time points during the cardiac cycle by using a powerful system of computers. This yields the values for pressure and velocity for each of the millions of elements. FFR can then be calculated for any point along the coronary artery tree by dividing the mean computed coronary artery pressure at the location of interest by the mean computed aortic pressure (72,73).

The current U.S. Food and Drug Administration (FDA)–approved postprocessing method is performed at a remote workstation and usually takes several hours per case. Once completed, the FFR value along any point in the coronary artery tree can be displayed on the final three-dimensional image. Additionally, the coronary artery tree is color-coded on the basis of a color scale corresponding to the FFR values (Fig 9). An FFR value less than 0.8 is considered abnormal (76). When added to the anatomic data from CCTA, the functional data provided by FFR CT improves its diagnostic accuracy secondary to improved specificity and positive predictive value (76,77). It is well known from the results of the FAME (FFR versus Angiography for Multivessel Evaluation) trial that of coronary stenoses that are anatomically classified as moderate (50%–69%) and severe (71%–90%), a significant number (two-thirds and one-fifth, respectively) are not hemodynamically significant (4).

**Figure 9a.**
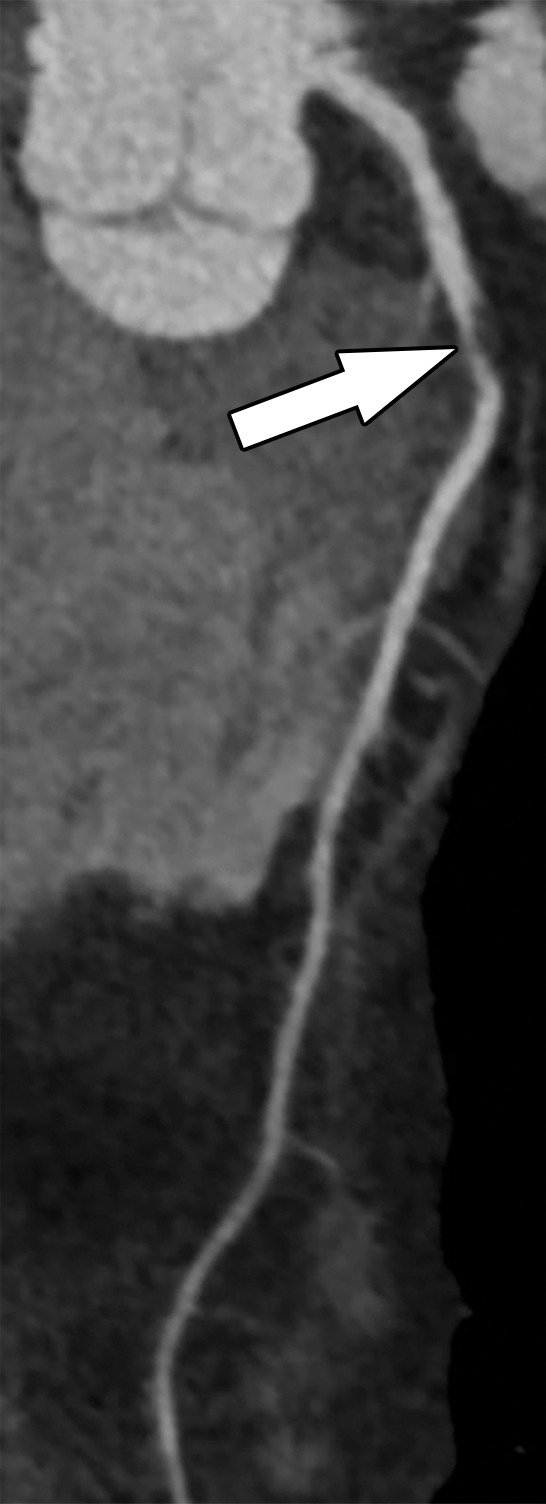
Abnormal exercise stress ECG result in a 52-year-old woman with type II diabetes mellitus. **(a)** Curved multiplanar reformatted CCTA image of the LAD coronary artery shows a severe (≥70%) mid LAD stenosis (arrow). **(b)** FFR CT image shows an FFR value less than 0.5 within the LAD distal to the mid LAD stenosis, compatible with a functionally significant coronary artery stenosis. **(c)** Coronary angiographic image during coronary catheterization before percutaneous coronary intervention again shows the mid LAD stenosis (arrow).

**Figure 9b.**
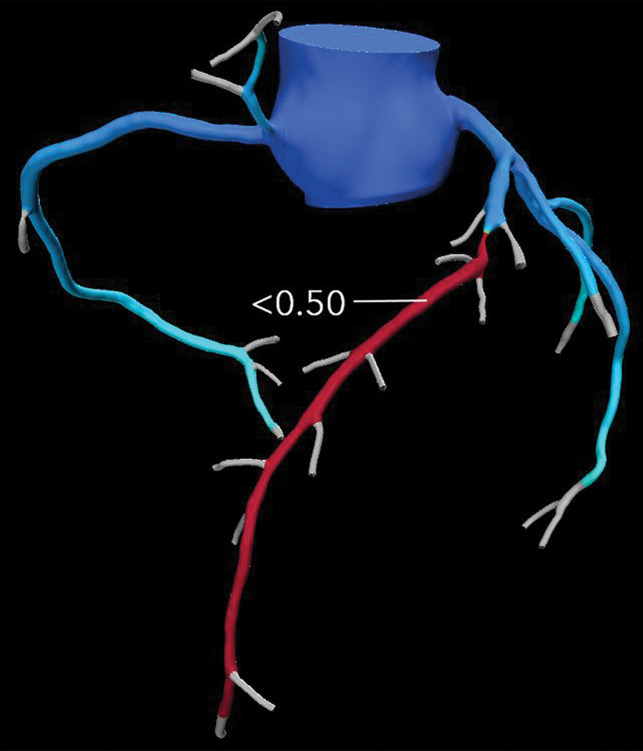
Abnormal exercise stress ECG result in a 52-year-old woman with type II diabetes mellitus. **(a)** Curved multiplanar reformatted CCTA image of the LAD coronary artery shows a severe (≥70%) mid LAD stenosis (arrow). **(b)** FFR CT image shows an FFR value less than 0.5 within the LAD distal to the mid LAD stenosis, compatible with a functionally significant coronary artery stenosis. **(c)** Coronary angiographic image during coronary catheterization before percutaneous coronary intervention again shows the mid LAD stenosis (arrow).

**Figure 9c.**
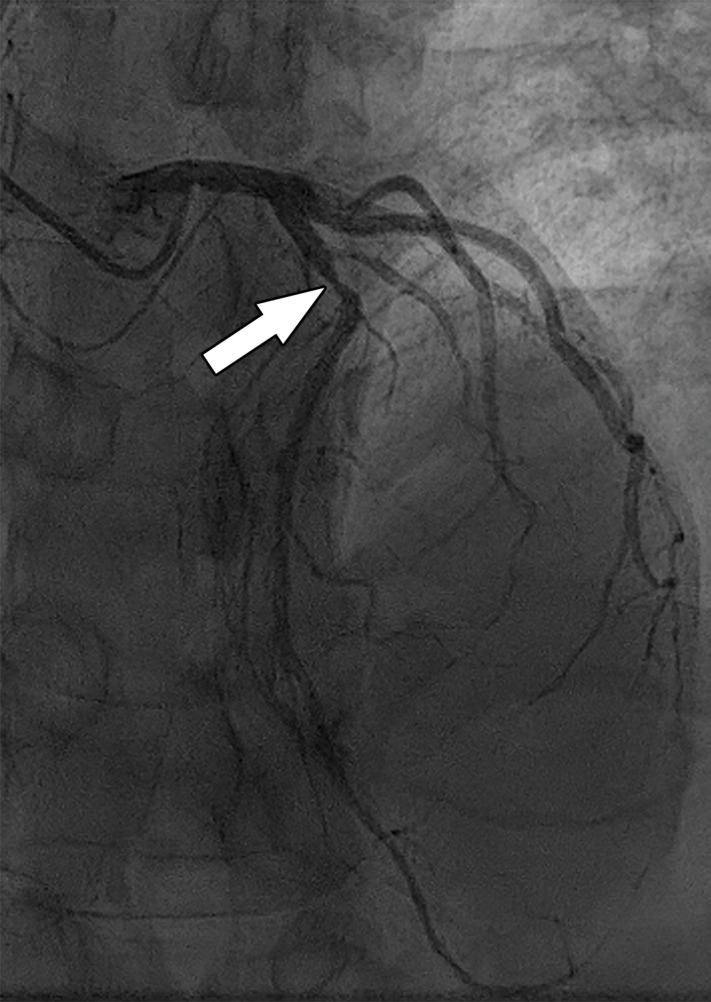
Abnormal exercise stress ECG result in a 52-year-old woman with type II diabetes mellitus. **(a)** Curved multiplanar reformatted CCTA image of the LAD coronary artery shows a severe (≥70%) mid LAD stenosis (arrow). **(b)** FFR CT image shows an FFR value less than 0.5 within the LAD distal to the mid LAD stenosis, compatible with a functionally significant coronary artery stenosis. **(c)** Coronary angiographic image during coronary catheterization before percutaneous coronary intervention again shows the mid LAD stenosis (arrow).

FFR CT functionally assesses moderate and severe coronary stenoses detected at CCTA, without any additional imaging or radiation exposure to the patient (Figs 10–11) (76,78). When directly compared with invasive FFR measurement as a reference standard, FFR CT performs well and has a sensitivity and specificity of 90% and 71%, respectively (56). Compared with CT stress perfusion imaging, FFR CT has similar diagnostic accuracy but does not involve additional radiation exposure from a second CT examination (64,77). Also similar to CT stress perfusion, FFR CT has best clinical utility in patients who have intermediate to high pretest probability (79).

**Figure 10a.**
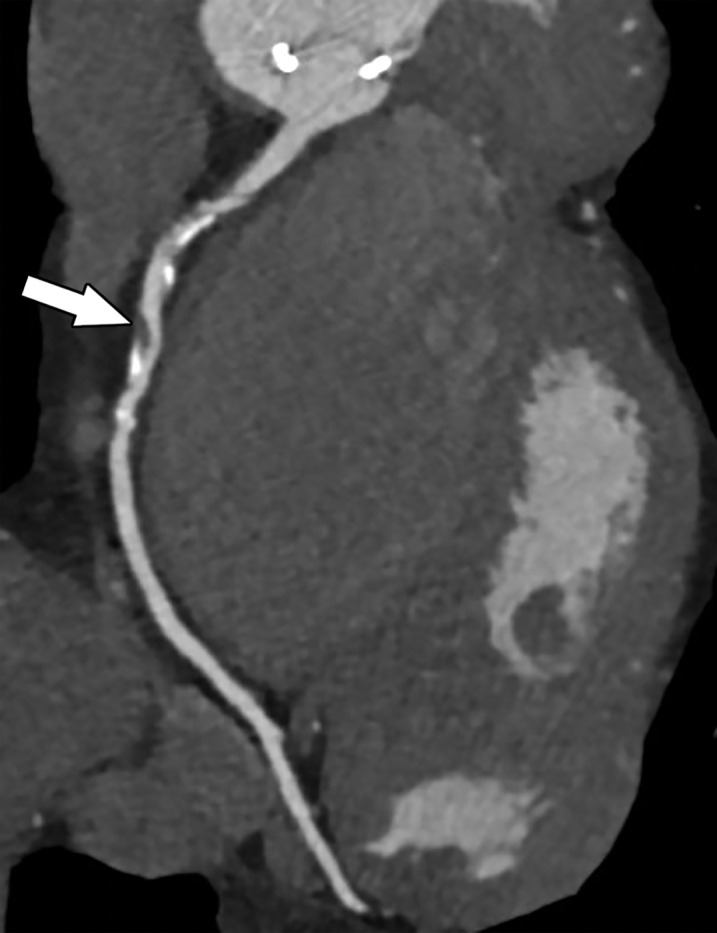
Exertional dyspnea in a 69-year-old man. **(a)** Curved multiplanar reformatted CCTA image of the right coronary artery (RCA) shows scattered calcified and noncalcified plaque within the proximal and mid vessel. There is an area of moderate (50%–69%) stenosis (arrow), secondary to mixed calcified and noncalcified plaque within the mid RCA. **(b)** FFR CT image shows an FFR value of 0.86 distal to the moderate RCA stenosis, consistent with no functional significance of the mid RCA stenosis.

**Figure 10b.**
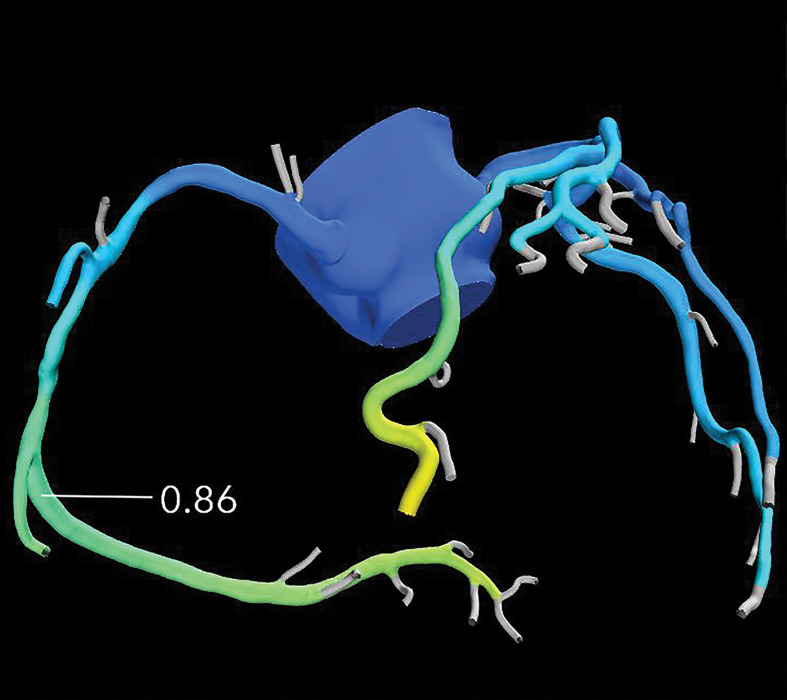
Exertional dyspnea in a 69-year-old man. **(a)** Curved multiplanar reformatted CCTA image of the right coronary artery (RCA) shows scattered calcified and noncalcified plaque within the proximal and mid vessel. There is an area of moderate (50%–69%) stenosis (arrow), secondary to mixed calcified and noncalcified plaque within the mid RCA. **(b)** FFR CT image shows an FFR value of 0.86 distal to the moderate RCA stenosis, consistent with no functional significance of the mid RCA stenosis.

**Figure 11a.**
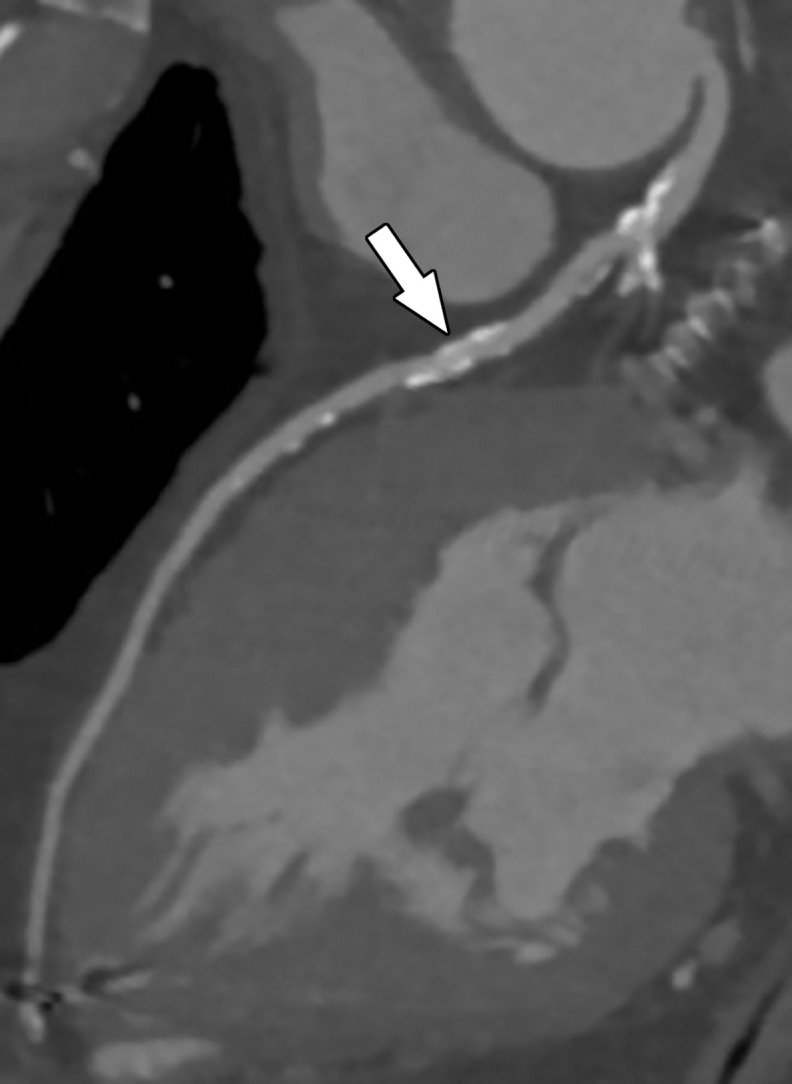
Referral for coronary CT as part of a preoperative evaluation in a 60-year-old man with a history of aortic valve replacement and complete heart block after dual-chamber pacemaker placement. **(a)** Curved multiplanar reformatted CCTA image of the LAD coronary artery shows a moderate (50%–69%) coronary stenosis (arrow) within the proximal LAD. **(b)** FFR CT image shows an FFR value of 0.72 distal to the moderate stenosis in the proximal LAD, compatible with a functionally significant coronary stenosis.

**Figure 11b.**
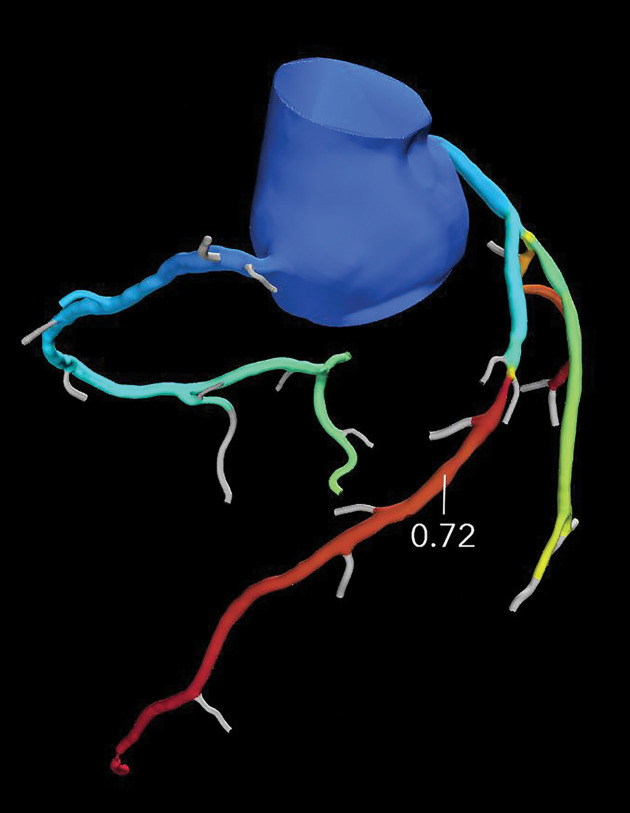
Referral for coronary CT as part of a preoperative evaluation in a 60-year-old man with a history of aortic valve replacement and complete heart block after dual-chamber pacemaker placement. **(a)** Curved multiplanar reformatted CCTA image of the LAD coronary artery shows a moderate (50%–69%) coronary stenosis (arrow) within the proximal LAD. **(b)** FFR CT image shows an FFR value of 0.72 distal to the moderate stenosis in the proximal LAD, compatible with a functionally significant coronary stenosis.

FFR CT has several additional advantages. No additional imaging, radiation exposure, or stress examination is needed to perform this technique; only the CCTA data are necessary. In addition, several previous trials including PLATFORM (Prospective Longitudinal Trial of FFRCT: Outcome and Resource Impacts), DISCOVER-FLOW (Diagnosis of Ischemia-Causing Stenoses Obtained Via Noninvasive Fractional Flow Reserve), and HeartFlowNXT (HeartFlow analysis of coronary blood flow using coronary CT angiography: NeXt sTeps) have found that the accurate identification at FFR CT of patients for percutaneous coronary intervention overall decreases health care costs while providing similar outcomes to those of invasive diagnostic methods (64). The major disadvantage of FFR CT is that it requires high-quality CT data. Thus, a study with motion or other artifacts may not be able to be processed correctly or at all. FFR CT availability remains limited, processing times are long, and it is precluded by renal failure. Additionally, FFR CT is not currently recommended if a coronary stent is present, in the setting of a coronary artery bypass graft (CABG), and in patients with congenital heart disease (77).

### PET Myocardial Perfusion Imaging

PET is a radionuclide imaging technique that relies on the detection of photons emitted from injected radiolabeled tracers. Photon generation occurs through the process of annihilation: positrons (emitted by the tracer) collide with an electron. Two photons are emitted in opposite directions and travel a short distance to the PET scanner. The PET scanner contains rings of detectors that convert this energy into an electrical signal, and it makes assumptions on coincidence detection and line of response. The photon counts from all of these lines of response are reconstructed into a map of radioactivity distribution (80–84). There are only two U.S. Food and Drug Administration–approved perfusion tracers: rubidium-82 (^82^Rb) and nitrogen 13 (^13^N) ammonia (^13^N-ammonia). ^13^N-ammonia is not nearly as commonly used as ^82^Rb given its need for cyclotron generation and a physical half-life of 9.9 minutes, which makes transport and/or shipping not feasible. Rubidium, which has an extremely short half-life of ~75 seconds, is produced from a strontium generator that can be used on site (80).

**Figure 12.**
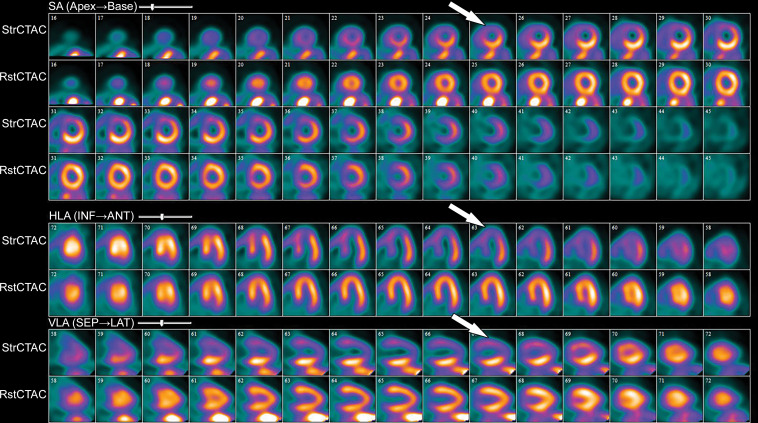
^82^Rb PET perfusion images show a severe perfusion defect (arrows) in the distribution of the LAD coronary artery at stress *(StrCTAC)* and normal perfusion at rest *(RstCTAC)*, compatible with ischemic myocardium. Metabolic images are not shown. *ANT* = anterior, *HLA* = horizontal long axis, *INF* = inferior, *SA* = short axis, *SEP* = septal, *VLA* = vertical long axis. Keys are the same for Figures 13, 18, and 19.

Similar to other stress perfusion techniques, both stress and rest images are obtained (Fig 12). Rest images are often obtained first at PET. Dynamic rest images are obtained with the heart at baseline and after the injection of the radionuclide, which depict the wash-in and washout of the radiotracer in the myocardium. Similarly, dynamic stress images are obtained during the administration of a vasodilator agent (adenosine, regadenoson, or dipyridamole, as described previously) and injection of the radionuclide. Time-activity curves of the blood and myocardium are extracted from the dynamic images, and kinetic modeling is used to quantify myocardial blood flow in milliliters per minute per gram, as well as the myocardial flow reserve (ratio of myocardial blood flow at stress to myocardial blood flow at rest) (81).

**Figure 13.**
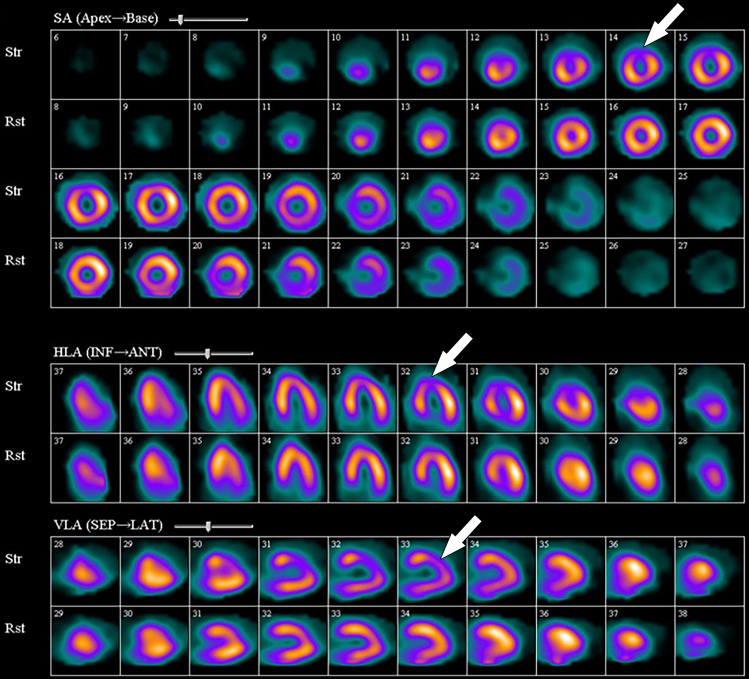
^99m^Tc–sestamibi images show a perfusion defect (arrows) at stress in the mid LAD coronary territory distribution along with transient ischemic dilatation, consistent with myocardial ischemia and possible multivessel disease.

The PET images can be interpreted qualitatively by comparing the stress and rest images and looking for areas of perfusion defect. The defect description should include the segments involved, the severity of the defect (mild, moderate, severe), as well as the amount of LV involved: small defect (5%–10% of the LV), medium (10%–20% of the LV), and large (>20% of the LV). Additionally, details of whether the defect is reversible (appears only on stress images secondary to ischemia) or fixed (appears both on stress and rest images secondary to an infarction) should be supplied.

A semiquantitative scoring system can score the severity of perfusion defects in each segment on the basis of the radiotracer count density: 0 = no defect; 1 = mildly reduced count; 2 = moderately reduced count; 3 = severely reduced count; 4 = absent activity. However, the major strengths of PET MPI are its ability to quantify myocardial blood flows (in milliliters per minute per gram) and to obtain a peak stress left ventricular ejection fraction (LVEF), which allows an LVEF “reserve” calculation, in addition to evaluation of wall motion abnormalities in areas with perfusion defects. Global myocardial blood flows and LVEF reserve (peak stress LVEF − resting LVEF) have both been shown to be independent predictors of future major adverse cardiac events. The ratio of myocardial blood flow at stress to myocardial blood flow at rest gives the myocardial flow reserve ratio. A ratio less than 1.5 is abnormal and indicates ischemia. An LVEF reserve of less than zero indicates an abnormal response to vasodilator stress (normal response is to increase with vasodilator stress) and provides incremental value (in addition to accounting for perfusion defects) in predicting major adverse cardiac events (80,85,86).

PET MPI has several advantages and disadvantages. PET MPI is robust with high accuracy and can be performed in patients with arrhythmias, advanced kidney disease, and pacemakers and/or defibrillators (80). PET MPI offers fully quantitative assessment of myocardial blood flow in milliliters per minute per gram of tissue and assesses myocardial function and myocardial metabolism (81).

Newer iterative reconstruction methods, improvements in detector technology, and three-dimensional acquisition have resulted in better spatial resolution (2 mm) and lower radiation doses (<2 mSv). With hybrid PET/CT scanners, low-radiation-dose noncontrast CT images of the heart are obtained before both stress and rest PET images for attenuation and scatter attenuation, which also allows screening and measurement of coronary artery calcium (81,87). It is also possible to combine coronary calcium scoring, CCTA, and PET into one examination. However, the PACIFIC (Prospective Comparison of Cardiac PET/CT, SPECT/CT Perfusion Imaging and CT Coronary Angiography With Invasive Coronary Angiography) trial did not find any increased value to this approach. The disadvantages of PET include radiation exposure from the radionuclides. However, it is less than that of CT and SPECT techniques (88).

Although originally used in research settings, PET MPI usage in clinical practice has increased over the years, secondary to increased radiotracer availability, new camera technologies, and data demonstrating its advantages over other MPI techniques. Unfortunately, despite its increased availability, PET still remains limited and is an expensive examination.

### SPECT Myocardial Perfusion Imaging

SPECT MPI is an older technique that relies on the detection of γ rays emitted from radionuclide agents. In comparison with other imaging methods, SPECT MPI remains the most commonly used noninvasive imaging study in clinical practice for several reasons: its widespread availability, its well-established standardized protocols, familiarity with the modality among providers, and the years of established data for diagnostic accuracy, allowing accurate risk stratification of patients.

As with other stress perfusion methods, SPECT MPI involves obtaining ECG-gated images at stress and rest. There are different protocols detailed in the American Society of Nuclear Cardiology guidelines, and either stress or rest can be performed first, although rest is typically performed first (39). If performed as a 2-day protocol (preferable for larger patients), the maximum dose of radionuclide is administered for both rest and stress portions of the examination. If done in 1 day, a smaller dose is administered for the rest images and a higher dose for the stress images. Some centers perform stress imaging first in lower-risk patients, with a rest study performed the following day only if stress perfusion defects are present. Stress imaging only is also performed in some centers. SPECT examinations can be performed by using exercise or a pharmacologic stress agent. The radionuclide agent is injected during peak exercise or at the time of peak vasodilation (adenosine, regadenoson, or dipyridamole protocols, as described previously). During exercise SPECT MPI, the examination continues until the patient becomes fatigued or develops symptoms warranting early termination of the test (severe chest pain or dyspnea, dizziness, arrhythmias, or hypotension). Rest images are obtained when the heart is at baseline and involve a second injection of the radionuclide agent (39,89). Low-radiation-dose CT can also be performed at SPECT MPI on hybrid SPECT/CT machines for attenuation correction purposes, which significantly improves soft-tissue artifacts. SPECT MPI radionuclide agents include thallium 201 (^201^Tl), technetium 99m (^99m^Tc)–sestamibi, ^99m^Tc-tetrofosmin, and ^99m^Tc-teboroxime. Technetium agents are preferred over ^201^Tl owing to its shorter half-life (less radiation dose to the patient) (89,90).

Stress and rest perfusion images are compared side by side to identify perfusion defects secondary to myocardial ischemia or infarction (Fig 13). The perfusion values within the LV are normalized to the area of myocardium with the highest count, which is the reason why balanced ischemia can sometimes be overlooked on SPECT MPI images. Similar to PET MPI, the location, severity, and size and whether the perfusion defect is reversible should be described. Stress-induced transient ischemic dilatation is a sign of severe multivessel CAD and should also be described.

The ECG-gated stress and rest images provide information on LV size, systolic function, and wall motion abnormalities. SPECT images suffer from several artifacts that mimic perfusion defects that should be noted if present. These artifacts are suggested by their typical locations and have normal LV wall motion in the area of the artifact on the ECG-gated images. Artifacts include soft-tissue attenuation from the breast affecting the anterior wall or diaphragm affecting the inferior wall (both of which can be confirmed by reviewing the raw images); apparent increased activity in the inferior wall secondary to subdiaphragmatic hepatobiliary excretion of technetium-labeled agents (which makes the remainder of the LV appear to have relatively low perfusion, as values in the LV are normalized to the area of highest count); motion artifact causing the heart to appear in different places on adjacent images (sometimes with associated artifactual defects), which warrants repeat imaging; and misregistration of SPECT and CT images. Normal apical thinning can cause apparent defects at the apex. However, these areas should have normal wall motion on the ECG-gated images. Additionally, left bundle branch block can cause an apparent defect in the septal wall, which is associated with paradoxical motion of the septum on the ECG-gated images (89,90).

The biggest advantage of SPECT is its widespread availability. Additionally, SPECT MPI can be performed in all patients. Unfortunately, it is a fairly long examination that suffers from higher radiation exposure, although newer technologies such as cardiac-specific solid-state detector cameras have lowered radiation doses to some degree. It also suffers from lower spatial resolution when compared with PET and has lower sensitivity and specificity when compared with MRI, PET, and CT methods. SPECT MPI examinations performed with exercise have decreased diagnostic accuracy if the patient cannot achieve a good heart rate response owing to physical or other limitations. Finally, SPECT MPI can overlook cases of balanced ischemia (56,91).

### Stress Echocardiography

Stress echocardiography is a widely available noninvasive imaging test used to risk stratify patients with suspected coronary disease. The stress echocardiographic examination observes the wall motion of the heart before, during, and after stress. Stress echocardiographic images are obtained immediately after treadmill exercise, during bicycle exercise, or during peak pharmacologic stress. Pharmacologic stress is obtained with dobutamine or a vasodilator (predominately dipyridamole). The minimum views obtained at stress echocardiography include parasternal long- and short-axis, apical four-chamber, and apical two-chamber views. The stress and rest echocardiographic images obtained are compared to assess for the development of a new or worsening wall motion abnormality that develops during stress, indicative of coronary ischemia. Wall motion abnormalities are graded as hypokinetic, akinetic, etc (92).

When directly compared with invasive FFR measurement as a reference standard, stress echocardiography has a sensitivity and specificity of 77% and 75%, respectively. Overall, this is similar to SPECT but below the performance of other noninvasive imaging modalities (MRI, PET, CT). The major advantages of stress echocardiography include its wide availability, low cost, lack of ionizing radiation, and portability. The biggest disadvantage is that images can be suboptimal, particularly in larger patients (56,92).

## Comparison of Noninvasive Imaging Modalities Assessing Physiologic Significance of Coronary Artery Stenosis

MRI stress perfusion, CT stress perfusion, FFR CT, PET MPI, SPECT MPI, and stress echocardiography all offer noninvasive functional assessment of coronary artery stenoses through various imaging technologies. However, only cardiac MR stress perfusion imaging, CT stress perfusion imaging, and PET MPI offer fully quantitative measurements of myocardial blood flow, which is considered the most accurate physiologic assessment of CAD and should not miss cases of balanced ischemia. Several studies and trials have performed a head-to-head comparison of these noninvasive perfusion techniques. Sensitivity, specificity, advantages, and disadvantages of all of these methods are summarized in Table 2 (56,70,88,93). Overall, the available evidence supports that the best noninvasive study to functionally assess coronary stenosis currently is cardiac MR stress perfusion imaging (sensitivity 90%, specificity 94%), closely followed by PET MPI (sensitivity 87%, specificity 84%) (56,93). Cardiac MRI has the additional benefits of aiding in the assessment of cardiac wall motion abnormalities, systolic function, and areas of fibrosis, thus allowing diagnosis of other cardiac diseases as well. Cardiac MRI involves no ionizing radiation. The major disadvantages of MRI are that its workflow can be cumbersome, it is not desirable to use gadolinium in patients with severe renal failure, and it is contraindicated by some implanted devices. PET MPI is not limited by the presence of renal failure or iatrogenic devices. However, it is expensive, has limited availability, involves radiation exposure to the patient (although it is less than CT techniques and SPECT), and is a longer examination (88).

**Table 2:**
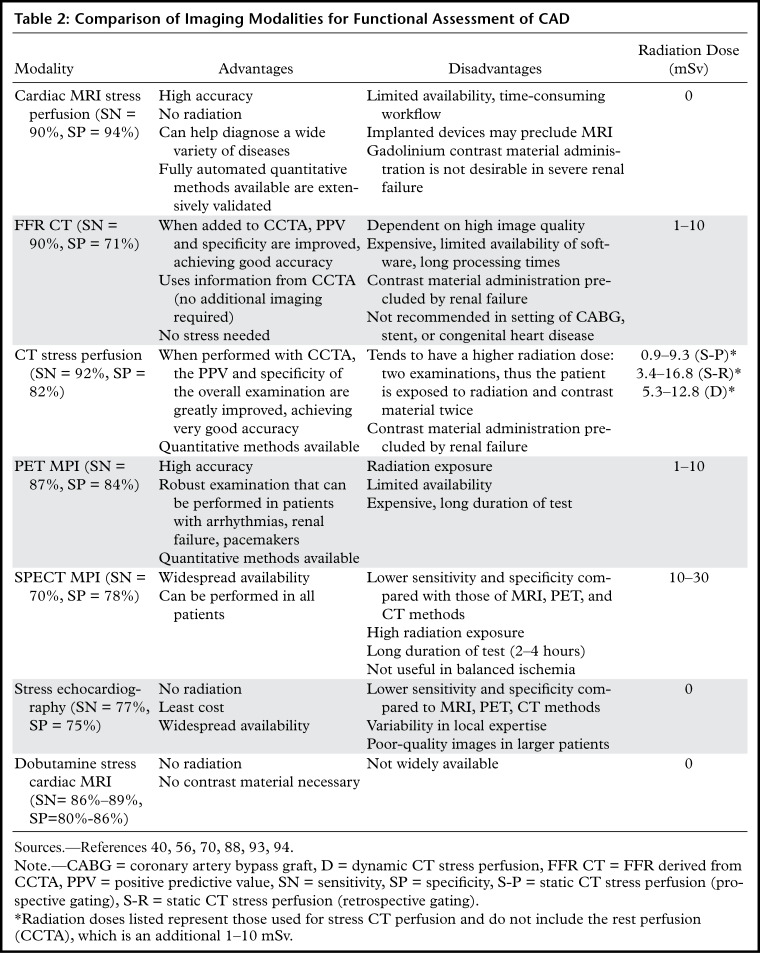
Comparison of Imaging Modalities for Functional Assessment of CAD

Both CT methods, CT stress perfusion imaging and FFR CT, rank just below MRI and PET methods, secondary to slightly lower specificities (specificity of 82% and 71%, respectively) but boast excellent sensitivity and negative predictive value (56,70). When interpreted in conjunction with tCCTA, the combined examination (either CCTA and FFR CT or CCTA and CT perfusion) has significantly improved specificity compared with CCTA alone, as well as high sensitivity. Both CT stress perfusion imaging and FFR CT involve radiation dose. CT stress perfusion has increased radiation dose compared with that at CCTA because two CT examinations are performed, while FFR CT does not increase the radiation exposure, as it is performed by postprocessing. FFR CT is highly dependent on image quality, is expensive, and has a long processing time. The use of CT is limited in patients with renal failure owing to the need for iodinated contrast material (70).

Compared with all of the other modalities, SPECT MPI and stress echocardiography overall have lower sensitivity, specificity, and diagnostic accuracy (56,88). However, both of these techniques are widely available compared with MRI, PET, and CT methods and can be performed in all patients. Stress echocardiography has the benefit of being low in cost as well. SPECT MPI is more widely available and less costly than PET MPI. However, SPECT has lower diagnostic accuracy compared with all other methods, and it also has the highest radiation dose to the patient (88).

Although some tests clearly have higher diagnostic performance, deciding on the best test for a patient may be daunting with so many choices available. Available data on current trends in cardiac imaging show that MRI, PET, and CT methods are performed far less often than SPECT and stress echocardiography, a finding that is strongly associated with the availability of these testing modalities. In fact, SPECT MPI remains the most commonly ordered test for reasons previously described. However, over the past several years, this trend is slowly starting to change, as the high-performance MRI, PET, and CT methods are becoming more widely available; the number of SPECT MPI and stress echocardiography examinations has been slowly decreasing, while the number of MRI, PET, and CT examinations has slowly been increasing (albeit fairly slowly) (95). Understanding the advantages and disadvantages of each noninvasive imaging test aids in determining which test is best for an individual patient. Ideally, the test chosen should have the highest performance possible, have the least amount of radiation exposure possible, and be available in the patient’s area. Availability of a test is often the limiting factor (with renal failure limiting availability of some options to the patient as well).

Based on the sensitivity and specificity of the tests (Table 2) there are two tiers of tests: high performance (cardiac MRI stress perfusion, PET MPI, FFR CT, and CT stress perfusion) and lower performance (SPECT MPI, and stress echocardiography). In addition, a negative CCTA examination safely excludes coronary disease, as concluded by several large trials (25–27). In terms of radiation exposure, MRI and stress echocardiography involve no ionizing radiation. FFR CT and PET MPI have lower radiation exposure, while CT stress perfusion and SPECT have a higher radiation dose. In terms of availability, SPECT and stress echocardiography have the widest availability. Additionally, MRI and CT methods are precluded in patients with severe end-stage renal failure.

**Figure 14.**
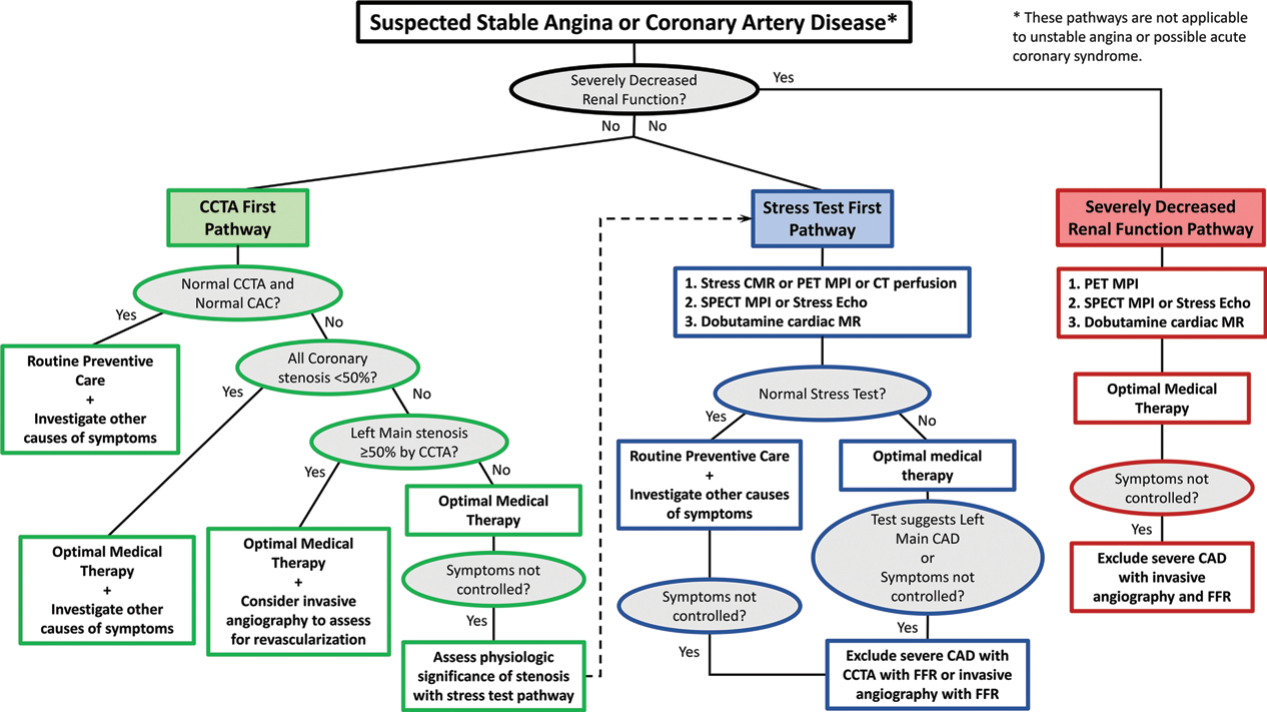
Flowchart shows an algorithm with recommendations to help select the best noninvasive imaging test for suspected stable angina or CAD. *CAC* = coronary artery calcium, *CMR* = cardiac MR, *Echo* = echocardiography.

A decision tree outlining three diagnostic pathways for assessing patients with suspected stable angina or CAD is presented in Figure 14. These pathways are not applicable to patients with unstable angina and acute coronary syndrome. As iodinated and gadolinium-based contrast material should be avoided in patients with severely reduced renal function, the first decision point is based on the presence of acute renal failure or chronic severely reduced renal function (estimated glomerular filtration rate <30 mL/min/1.73 m^2^). The CCTA-first pathway should be favored in patients with low pretest probability of CAD for several reasons: *(a)* CCTA works well in normal or near-normal coronary arteries, *(b)* CCTA has excellent negative predictive value safely excluding CAD, and *(c)* a normal CCTA examination and normal coronary calcium score suggest excellent prognosis. As such, if the CCTA and coronary calcium score are both normal, the patient should receive routine preventive medicine, and physicians should consider alternative diagnoses.

If the CCTA examination is abnormal but all stenoses are less than 50% in severity, the patient should receive optimal medical therapy for CAD, but physicians should also pursue alternative diagnoses since a stenosis less than 50% is unlikely to explain stable angina. If the CCTA images depict a greater than or equal to 50% left main coronary artery stenosis, the patient should receive optimal medical therapy, and their physicians should consider referring the patient for invasive angiography and possible revascularization. Patients with severe stenosis not involving the left main coronary artery can be treated with optimal medical therapy initially. Failure to control symptoms with optimal medical therapy can be because of severe physiologically significant CAD or because the severity of stenosis detected at CCTA was not physiologically significant and there is an alternative reason for the patient’s angina. The specificity of CCTA without CT FFR is described earlier in this article. Thus, switching at this point to the stress test pathway is more reasonable than repeating CCTA or going straight to invasive angiography.

The stress test–first pathway (Fig 14) should be favored initially in older patients and in patients with a higher pretest likelihood of CAD, although a precise cut point has not been determined, and this recommendation is made on clinical experience. It is also the most appropriate pathway for patients with coronary stents. The stress test–first strategy needs to be retained because most current clinical practices start CAD assessment with a stress test and it will be hard to move practitioners from that approach, and realistically the approach works well. Furthermore, most countries, including the United States and the United Kingdom, are not prepared to replace millions of SPECT and stress echocardiography examinations with CCTA in the near future.

The selection order of stress tests is organized by tiers of relatively equivalent diagnostic accuracy. First-tier tests have the highest accuracy and include cardiac MR stress perfusion imaging, PET MPI, and CT stress perfusion. The second-tier tests include SPECT MPI and stress echocardiography. The third-tier test is dobutamine cardiac MR. The second-tier tests cannot be eliminated owing to limited availability of the advanced first-tier cardiac imaging methods, expense, relatively small differences in diagnostic performance, and other considerations. Dobutamine stress cardiac MR is listed as a third-tier test despite good diagnostic accuracy because there are relatively few centers in the United States and most countries in the world that perform a significant number of these tests. A negative stress test should trigger routine preventive care, and the physician should investigate other causes of the symptoms. If the stress test result is abnormal, optimal medical therapy should be prescribed. Failure of optimal medical therapy in either part of the pathway could be due to a noncoronary cause of the symptoms or CAD so severe that revascularization might be needed. At this point, CCTA with FFR (FFR CT) or invasive angiography with FFR could help resolve the residual clinical questions related to CAD.

Finally, the severely decreased renal function pathway (Fig 14) omits CCTA and stress perfusion cardiac MRI because they both require the administration of contrast material that should be avoided in these patients. The residual tests are ranked in tiers as described in the stress test–first pathway, and the logic of the remaining parts of the pathway follows a similar rationale. Dobutamine stress cardiac MRI has one additional weakness in this pathway, as one generally avoids administration of gadolinium contrast material in these patients, and this means that LGE imaging of MI is not performed.

## Myocardial Infarction

Acute disruption of coronary artery blood flow, which most commonly occurs owing to plaque rupture or erosion, may result in prolonged myocardial ischemia and subsequent MI. Less common causes of MI include coronary vasospasm, vasculitis, embolism, coronary artery dissection, or various iatrogenic injuries. Cardiac MRI with gadolinium contrast material remains the standard test for assessment of myocardial viability, which was first established in a landmark article by Kim et al (8) in 1999. PET can also help accurately detect MI. SPECT is more limited as it can find matched perfusion defects suggestive of MI but cannot differentiate an MI from hibernating or stunned myocardium as reliably as can MRI and PET. Hibernating myocardium refers to a chronic state of ischemia that results in decreased regional function that may recover function after successful coronary revascularization. Stunned myocardium is a prolonged regional dysfunction on the order of minutes to days after transient stress-induced ischemia, which recovers without revascularization. CT is not a standard test used to assess for an MI. However, MI can often be incidentally detected on CT images.

### Cardiac MRI

Myocyte death increases the extracellular volume in the infarcted myocardium, resulting in delayed wash-in and washout of gadolinium, which subsequently shortens its T1 relaxation time so that it appears bright relative to normal myocardium on T1-weighted inversion-recovery images obtained 10–20 minutes after a 0.1–0.2 mmol/kg intravenous dose of gadolinium contrast material on cardiac MR images. These images, referred to as LGE images, delineate the amount of infarcted myocardium (96,97). Currently, only gadobutrol is approved at a dose of 0.1 mmol/kg to assess myocardial perfusion (stress, rest) and MI (LGE imaging), but other gadolinium contrast material, as well as higher doses of gadolinium, is used.

The conventional LGE method is the segmented inversion-recovery LGE method, which is an inversion-recovery sequence with a segmented FLASH readout. The segmented acquisition means that a fraction of the raw data are obtained during each heartbeat in diastole with consistent inversion time after the QRS complex of the ECG. Most protocols complete data acquisition over about 8–12 cardiac cycles during a breath hold. In this method, an inversion time is manually selected by the technologist to null the normal myocardium, resulting in extremely low signal intensity of the normal myocardium (98).

Segmented LGE images can be reconstructed as both magnitude and phase-sensitive images. Magnitude reconstruction is the most common method used for reconstructing MR images, but its appearance is highly sensitive to the selected inversion time. Phase-sensitive reconstruction produces consistently good image quality even if the technologist’s selected inversion time is slightly off (58). Phase-sensitive reconstruction uses the data needed to produce a magnitude LGE image but also uses a reference image obtained on the second heartbeat after the inversion pulse to retain positive and negative signals in the complex data domain. As with velocity-encoded phase contrast–enhanced MRI, phase-sensitive inversion recovery maintains positive and negative signals relative to the signal intensity of the ideal null point. This allows retrospective adjustment of window and level to display LGE images that were acquired with an imperfect inversion time in an appearance that is quite similar to the properly nulled magnitude image (99). However, one should review both the magnitude and the phase-sensitive inversion-recovery images. If the inversion time selected to null the myocardium is too far off on the magnitude images, then the phase-sensitive images also become unreliable, despite appearing to be of diagnostic quality.

**Figure 15.**
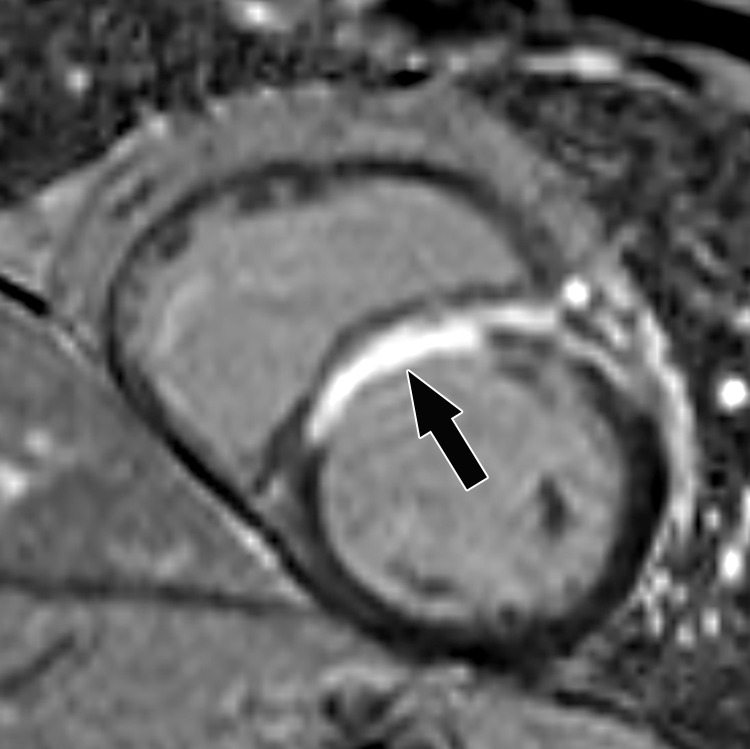
Subendocardial enhancement in a 44-year-old man with a history of CAD, MI, and tobacco use who was referred for cardiac MRI to assess MI size. Short-axis LGE cardiac MR image of the LV shows subendocardial-based enhancement, with about 75% transmural extent within the basal septal wall (arrow), as well as some extension into the adjacent anterior wall. The transmural extent of enhancement is greater than 50%, which indicates a lower likelihood of recovery with revascularization.

The most characteristic finding of MI on cardiac MR images is involvement of the subendocardial layers of myocardium (ie, adjacent to the LV cavity), with a variable transmural extent generally graded per the following scale: none, 1%–25%, 26%–50%, 51%–75%, 76%–99%, and 100% (Fig 15). Description of the MI detected on LGE images should include the segments involved and the transmural extent of enhancement. Accurate delineation of MI is important when deciding whether to revascularize. MI that has greater than 50% transmural extent within a segment has a much lower likelihood of recovery after revascularization (100). However, one must use caution in interpreting the amount of myocardial scar in the setting of acute myocardial injury. Myocardial edema and inflammation lead to swelling of the acutely injured myocardium, as well as adjacent viable areas of myocardium (101). Microvascular obstruction (no reflow areas secondary to microthrombi in the infarcted tissue) or intramyocardial hemorrhage in areas of infarction appear as areas of low signal intensity on early gadolinium-enhanced images and should be noted. Areas of microvascular obstruction are associated with poorer prognosis (97).

Additional findings of MI include wall motion abnormalities, thinning of the myocardial wall, and decreased ejection fraction. Wall motion abnormalities with no corresponding LGE correspond to stunned or hibernating myocardium that may recover without and with revascularization, respectively. More recently, precontrast and postcontrast (before and after the administration of contrast material, respectively) T1 maps can be used to generate extracellular volume (ECV) maps. ECV maps display the percentage of ECV in the myocardium on a pixel-wise level. An ECV greater than 29% indicates abnormally increased extracellular volume (dependent on the methods used), which could be secondary to fibrosis from MI or other processes, edema, amyloidosis, etc (102).

**Figure 16.**
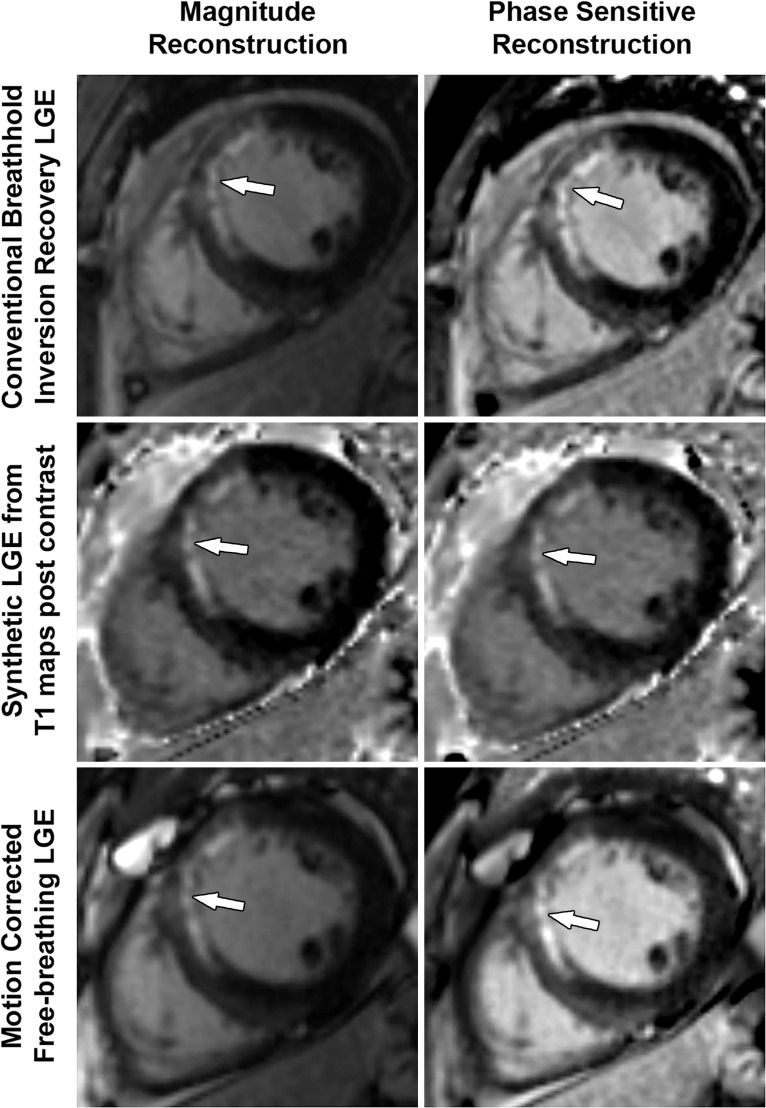
Comparison of LGE images of an MI in the LAD territory in a 48-year-old man with an LAD occlusion, obtained by three different acquisition methods and two different image reconstruction methods, shows the similarity of diagnostic information. Left column: conventional magnitude image reconstruction. Right column: phase-sensitive inversion-recovery reconstruction. Top row: conventional breath-hold segmented inversion recovery. Middle row: synthetic inversion-recovery LGE images generated from postcontrast T1 maps. Knowing the T1 value on a pixel-by-pixel basis allows estimation of image intensity on a typical inversion-recovery LGE image at any inversion time, including the optimal inversion time for nulling normal myocardium. Bottom row: motion-corrected free-breathing LGE method. The image quality of the synthetic and motion-corrected methods is good, and they have the advantage that they can be performed without breath hold. However, they have lower resolution than the conventional breath-hold segmented inversion-recovery LGE images.

The conventional segmented inversion-recovery method provides high-spatial-resolution LGE images under ideal conditions but has two major weaknesses. Its quality is easily degraded if there is motion artifact resulting from imperfect breath holds or if there is an incorrect inversion time. Newer LGE methods offer solutions to these problems. These methods include single-shot inversion-recovery LGE, postcontrast T1 mapping, synthetic inversion-recovery LGE methods derived from T1 mapping, and motion-corrected free-breathing LGE (103–106). All of these methods use a motion correction algorithm, except for single-shot LGE. Synthetic inversion-recovery methods do not require selection of an inversion time. However, when compared with the conventional segmented inversion-recovery method, all of these methods, although diagnostic, are slightly inferior in image quality (Fig 16). In patients who cannot hold their breath, the newer methods may be superior to the conventional LGE images.

### Computed Tomography

Although ECG-gated CT of the heart is not recommended to assess for myocardial viability, findings of MI are frequently identifiable and incidentally found on both cardiac CT and nongated thoracic and abdominal CT images. Thus, familiarity with the appearance of MI at CT remains important. The main findings include a regional area of hypoperfusion, focal myocardial thinning, and myocardial calcifications (Fig 17) (107). Fatty metaplasia, a complication seen in chronic MI, can also be visualized easily on CT images and is discussed with other MI complications in this article. On retrospectively-gated cardiac CT images, LV wall motion abnormalities and systolic function can also be assessed. If dual-energy CT is performed, iodine maps can be created to look for areas of no iodine uptake within the myocardium that correspond to MI (108).

**Figure 17a.**
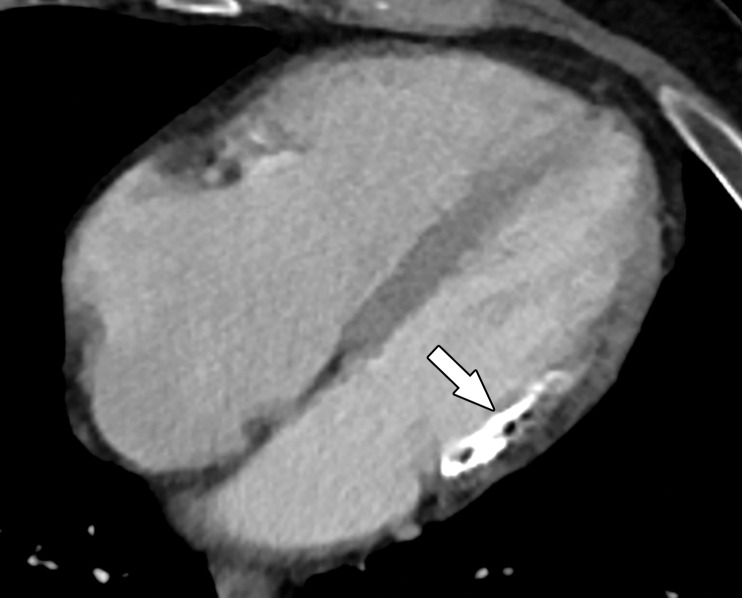
Four-chamber **(a)** and short-axis **(b)** CT images in a 65-year-old man show an area of hypoenhancement and calcification within a thinned nonenhancing basal inferolateral wall (arrow), compatible with a chronic MI. This MI was incidentally detected on a routine nongated contrast-enhanced chest CT image obtained for chronic dyspnea.

**Figure 17b.**
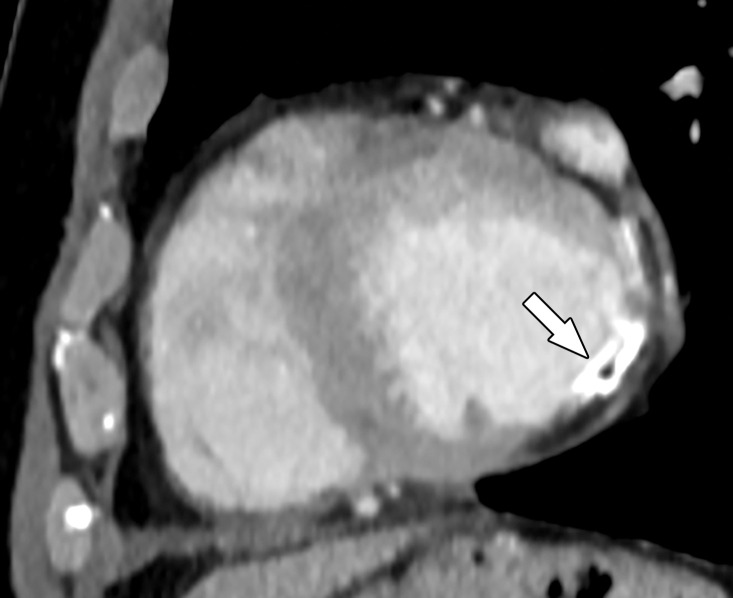
Four-chamber **(a)** and short-axis **(b)** CT images in a 65-year-old man show an area of hypoenhancement and calcification within a thinned nonenhancing basal inferolateral wall (arrow), compatible with a chronic MI. This MI was incidentally detected on a routine nongated contrast-enhanced chest CT image obtained for chronic dyspnea.

Similar in concept to LGE images obtained during cardiac MRI, a delayed CT image obtained approximately 10 minutes after iodinated contrast material administration that can detect MI has been recently described. However, continued work is needed to further validate this method. Performing this delayed CT examination also results in additional radiation exposure to the patient (63).

### PET and SPECT

**Figure 18.**
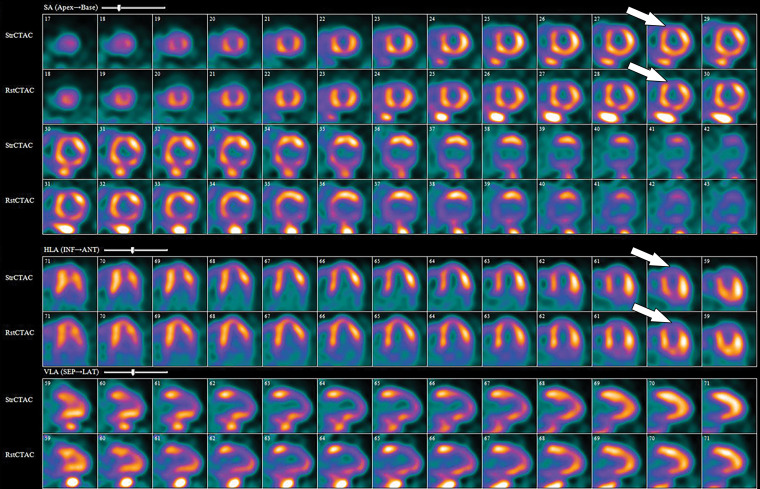
^82^Rb PET perfusion images show a fixed perfusion defect on both stress *(StrCTAC)* and rest *(RstCTAC)* images (arrows) within the anterior wall. This matched pattern is compatible with MI. Metabolic images are not shown.

**Figure 19.**
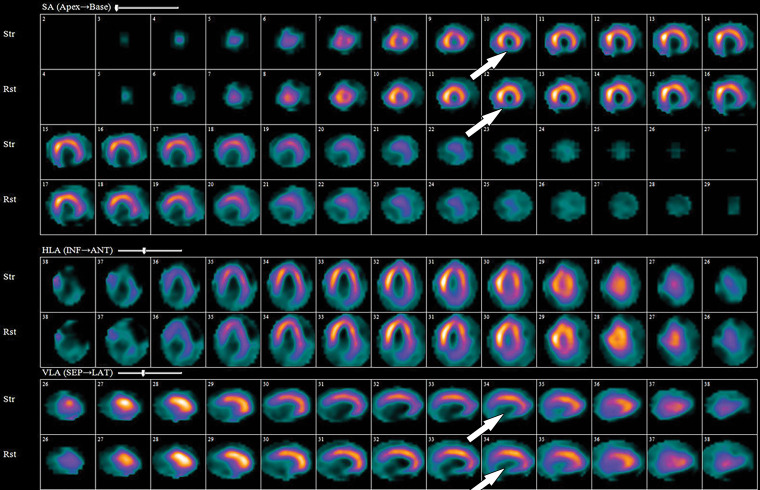
^99m^Tc-sestamibi images show a fixed defect in the inferior wall (arrows), which is highly suggestive of a right coronary artery territory MI. *Rst* = rest, *Str* = stress.

PET and SPECT MPI procedures and interpretation are detailed earlier in this article. Both PET and SPECT examinations can help identify matched defects on stress and rest images, although SPECT suffers from lower spatial resolution. These matched defects may represent MI or hibernating myocardium (Figs 18–19). Hibernating myocardium is the result of a chronic state of ischemia that results in decreased regional function and perfusion that may recover function after successful coronary revascularization. An advantage of PET is the ability to obtain metabolic images that can be compared with the PET perfusion images. Myocardial metabolic tracers include fluorine 18 (^18^F) fluorodeoxyglucose and carbon-11 (^11^C) acetate. Metabolic activity within the matched defect on PET perfusion images is consistent with viable hibernating myocardium, which would likely improve after revascularization (39,80,82). Combined PET/MRI has shown that metabolic activity observed at PET correlates well with the transmural extent of LGE at MRI (82).

## Imaging Complications of MI

Complications of MI are best identified at echocardiography, cardiac MRI, and cardiac CT. These include LV aneurysm and pseudoaneurysm, LV thrombus, fatty metaplasia of chronic MI, ischemic ventricular septal defect, intramyocardial hematoma in the setting of an acute MI, acute mitral regurgitation, and Dressler syndrome.

Left ventricular aneurysm and pseudoaneurysm infrequently complicate MI, but differentiating true aneurysm from pseudoaneurysm is important because of their different management and prognosis. A true aneurysm is a wide-mouthed outpouching of the LV that involves all three layers of myocardium. The myocardium is thinned, akinetic or dyskinetic, and enhances on LGE images (Fig 20). True aneurysms are fairly stable and can calcify over time (109). Because true LV aneurysms are often severely hypokinetic or akinetic, LV thrombus formation is likely (Fig 21). LGE imaging is the best technique to help detect LV thrombus (sensitivity, 88%; specificity, 99%) and can help detect small thrombi missed at cine MRI and echocardiography (110). In comparison with a true aneurysm, a pseudoaneurysm is much more ominous and requires surgical repair. A pseudoaneurysm is an LV rupture that is contained by scar tissue or pericardium. At imaging, it appears as an outpouching arising from the LV that typically has a narrow neck (Fig 22) (97,109).

**Figure 20a.**
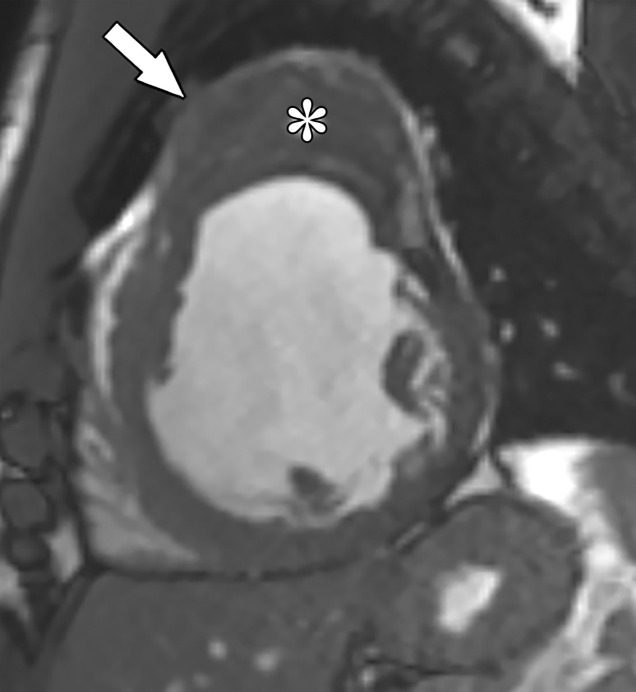
True aneurysm in a 42-year-old man with cardiomyopathy of unknown cause who was referred for further evaluation. **(a)** Short-axis image from SSFP cine MRI shows a wide-mouthed outpouching (arrow), compatible with an aneurysm, arising from the anterior wall of the LV and containing intermediate-signal-intensity material (*) within it. **(b)** Short-axis phase-sensitive segmented inversion-recovery LGE image shows diffuse enhancement of the aneurysm wall (arrow), compatible with infarcted myocardium, as well as a low-signal-intensity thrombus within the aneurysm.

**Figure 20b.**
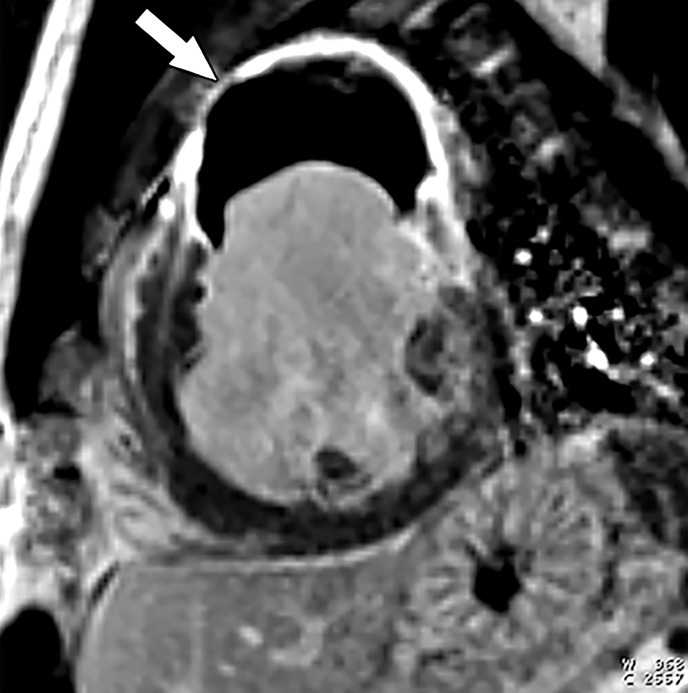
True aneurysm in a 42-year-old man with cardiomyopathy of unknown cause who was referred for further evaluation. **(a)** Short-axis image from SSFP cine MRI shows a wide-mouthed outpouching (arrow), compatible with an aneurysm, arising from the anterior wall of the LV and containing intermediate-signal-intensity material (*) within it. **(b)** Short-axis phase-sensitive segmented inversion-recovery LGE image shows diffuse enhancement of the aneurysm wall (arrow), compatible with infarcted myocardium, as well as a low-signal-intensity thrombus within the aneurysm.

**Figure 21.**
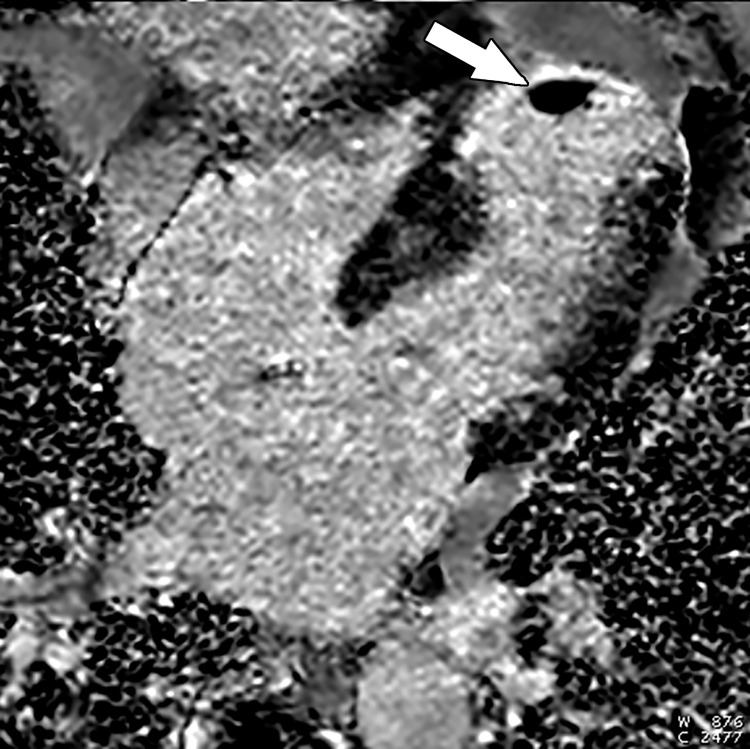
Left ventricular apical aneurysm with an apical thrombus in a 69-year-old woman with a history of MI. Four-chamber phase-sensitive inversion-recovery LGE image shows a left ventricular thrombus (arrow) in an aneurysmal infarcted left ventricular apex. Note the transmural enhancement of the left ventricular apex, compatible with MI.

**Figure 22.**
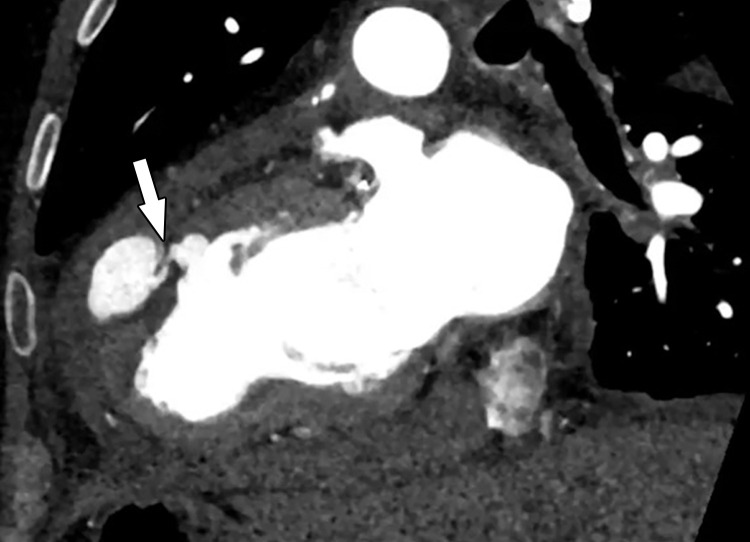
Pseudoaneurysm in a 61-year-old man with worsening chest pain 2 weeks after coronary artery bypass surgery who was referred for CT angiography for further evaluation. CT angiographic image shows an outpouching of contrast material with a narrow neck (arrow) arising from the LV, compatible with a pseudoaneurysm.

**Figure 23.**
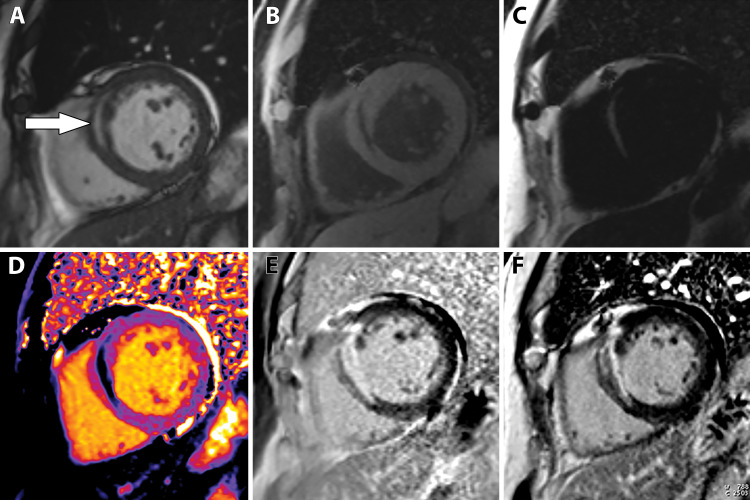
Fatty metaplasia of chronic MI in a 67-year-old man with a history of MI many years prior. Cardiac MR images show midwall fatty metaplasia within a chronic near-transmural MI of the basal septum. Short-axis image from cine SSFP MRI, *A,* shows midwall hyperintensity within the basal anteroseptum (arrow). Water separation, *B,* and fat separation, *C,* images confirm that the midwall abnormality is fat. T1 map, *D,* shows a midwall area in the basal anteroseptum that has a T1 value consistent with that of fat. Early, *E,* and late, *F,* gadolinium enhancement images show a near-transmural MI involving the basal anteroseptal wall.

Fatty metaplasia of chronic MI is the sequela of the infarct healing and/or remodeling process. It is fairly common and increases the risk of ventricular arrhythmias (97,111). It is easily detected on both cardiac MR and CT images as an area of curvilinear fat within the area of MI. At cardiac MRI, it is best identified with native T1 mapping, chemical shift artifact on cine MRI imaging, black blood imaging with and without fat suppression, or ECG-gated multi-echo Dixon fat-water separation imaging (Fig 23) (111).

**Figure 24.**
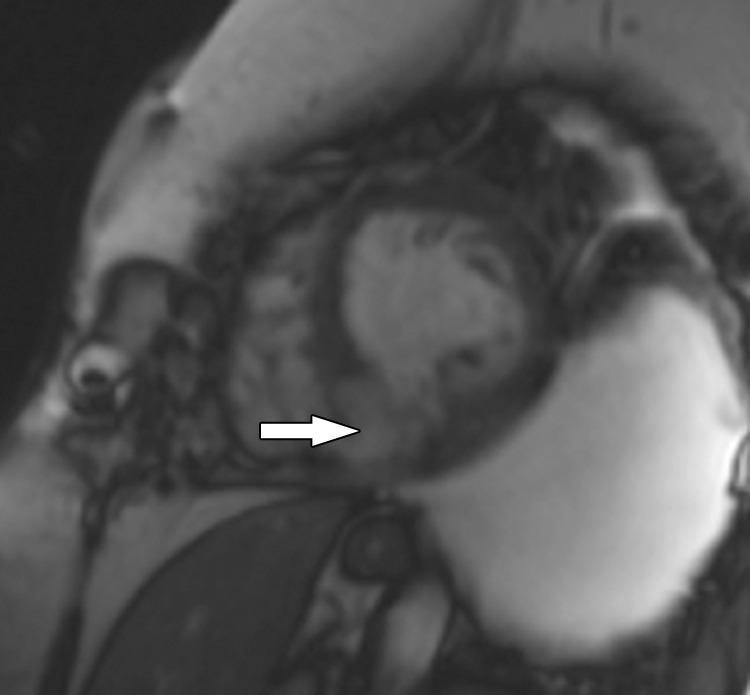
Inferior MI in a 61-year-old woman who developed shortness of breath. Echocardiogram (not shown) depicted an ischemic ventricular septal defect in the location of a prior MI, and the patient was referred for cardiac MRI for further evaluation. Short-axis image from SSFP cine MRI shows an ischemic ventricular septal defect (arrow) between the left and right ventricles at the site of the MI. Supplemental MRI cine clips of the short axis and left ventricular outflow show a flow jet from the LV into the right ventricle through the ischemic ventricular septal defect (Movies 1, 2).

Ischemic ventricular septal defect (Fig 24) is a fairly uncommon complication, with an incidence of 0.17% and which accounts for approximately 5% of acute MI deaths. Approximately half of these patients die within the first week, and even after surgery approximately 40% do not survive. Risk factors include hypertension and single vessel occlusion resulting in a large MI. Ischemic ventricular septal defects associated with inferior wall MI tend to have an associated basal inferior wall aneurysm as well. These patients present with a new holosystolic murmur and new chest pain, dyspnea, or cardiogenic shock. Treatment is surgery, but even after surgery their prognosis is poor. The ischemic ventricular defect can be detected at echocardiography and cardiac MRI and CT. Left to right flow between two ventricles may be observed with Doppler US and velocity-encoded phase-contrast imaging. Cardiac MRI cine imaging also shows flow from the left to right ventricle across the defect (97,112).

**Movie 1:** Short-axis MR cine clip shows a flow jet from the LV into the right ventricle through the ischemic ventricular septal defect.

**Movie 2:** Left ventricular outflow cine clip shows a flow jet from the LV into the right ventricle through the ischemic ventricular septal defect.

Intramyocardial hematoma is an exceedingly rare life-threatening complication of an MI that is often first visualized on an echocardiogram but is best characterized with cardiac MRI and/or CT (Fig 25). Rupture of the infarcted myocardium results in intramyocardial hematoma formation that expands and dissects through the infarcted myocardium. Cardiac MRI LGE imaging will depict an intramyocardial expansile area of low signal intensity, compatible with the intramyocardial hematoma, within the enhancing infarcted ventricular wall. CT images may depict contrast-enhanced blood within an expanded myocardial wall (113,114).

**Figure 25a.**
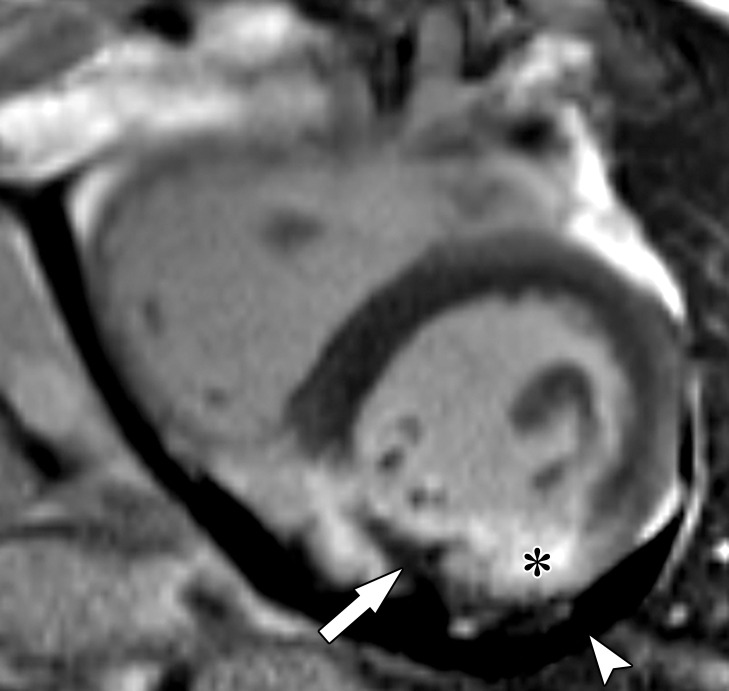
Intramyocardial hematoma in a 60-year-old man with a history of MI. Recent echocardiographic findings (not shown) were suggestive of expanding posterior wall intramyocardial hemorrhage and dissecting intramyocardial hematoma after an acute MI.**(a)** Short-axis phase-sensitive inversion-recovery LGE image shows a transmural MI (*), with an area of nonenhancing intramyocardial hematoma (arrow). Pericardial effusion is also depicted (arrowhead).**(b)** Coronal CT image shows contrast material infiltration into the myocardial wall (arrow). Moderate pericardial effusion (*) is present.*LA* = left atrium,*RA* = right atrium.

**Figure 25b.**
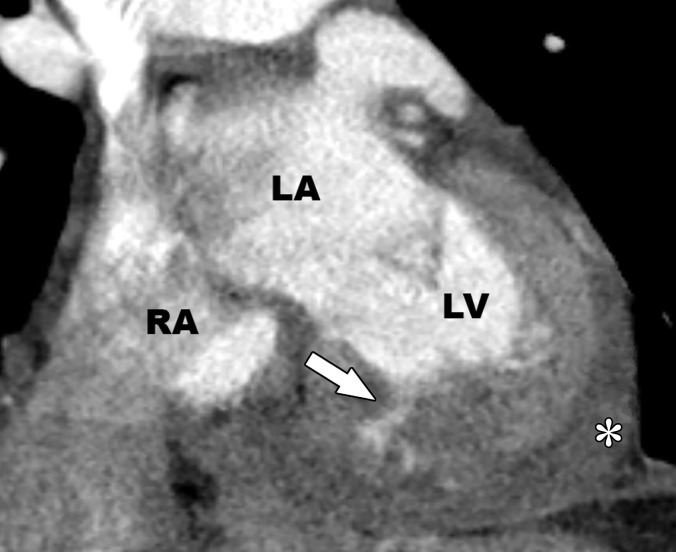
Intramyocardial hematoma in a 60-year-old man with a history of MI. Recent echocardiographic findings (not shown) were suggestive of expanding posterior wall intramyocardial hemorrhage and dissecting intramyocardial hematoma after an acute MI.**(a)** Short-axis phase-sensitive inversion-recovery LGE image shows a transmural MI (*), with an area of nonenhancing intramyocardial hematoma (arrow). Pericardial effusion is also depicted (arrowhead).**(b)** Coronal CT image shows contrast material infiltration into the myocardial wall (arrow). Moderate pericardial effusion (*) is present.*LA* = left atrium,*RA* = right atrium.

Ischemic mitral regurgitation is an uncommon emergency after an acute MI that has two major mechanisms: abrupt papillary muscle rupture and papillary muscle dysfunction following acute MI, both of which worsen the prognosis of the acute MI. Ischemic mitral regurgitation tends to occur with an inferior wall infarction that affects the posteromedial papillary muscle. Papillary muscle dysfunction is more common than papillary muscle rupture, but both are rare. Ischemic mitral regurgitation commonly manifests as acute pulmonary edema, sometimes localized to the right upper lobe, which may be the first sign noticed on a chest radiograph. The ischemic mitral regurgitation can be visualized on an echocardiogram as well as on a cardiac MR velocity-encoded phase-contrast image. Treatment is valvular surgery (112,115).

**Figure 26.**
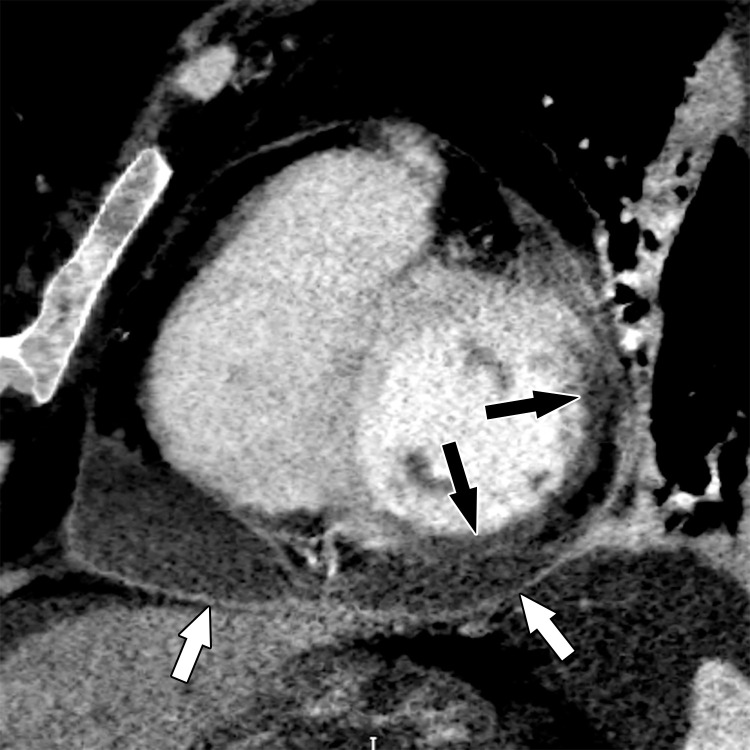
Postmyocardial infarction pericarditis (Dressler syndrome) in a 58-year-old woman who returned to the emergency department for chest pain 12 days after discharge following an acute MI. Short-axis 4-mm-thick minimum intensity projection (MinIP) image shows transmural hypoattenuation of the anterolateral, inferolateral, and inferior segments at the midcavity level, owing to recent proximal left circumflex coronary artery territory infarction (black arrows). A moderate-size pericardial effusion is depicted, with areas of pericardial enhancement (white arrows), consistent with pericarditis. The patient’s symptoms dramatically improved after initiation of anti-inflammatory medications.

Dressler syndrome is another MI-related complication seen in up to 5% of patients. The syndrome manifests with low-grade fever, pericardial rub, and pleuritic chest pain after an MI, and it is believed to be secondary to an autoimmune reaction to the myocardial necrosis that occurs 2–10 weeks after the infarction. Both cardiac MR and CT images show thickening of the pericardium and pericardial effusion acutely (Fig 26). LGE images from cardiac MRI show enhancement of the pericardium in chronic cases (116).

## Conclusion

Management of ischemic heart disease, which remains one of the leading causes of death worldwide, is now best guided by the physiologic significance of coronary artery stenosis. Unrecognized MI is another clear indicator of physiologically significant CAD. CCTA remains an effective first-line examination to exclude CAD in patients with low pretest probability but cannot assess the physiologic significance of a coronary stenosis without additional functional analysis of the coronary stenosis. Advances in multiple noninvasive imaging techniques, in particular quantitative capabilities in MRI, CT, and PET, allow accurate detection and functional assessment of CAD in patients with intermediate to high pretest probability and have enabled these noninvasive imaging methods to assume a key role in guiding therapeutic intervention. MI may be identified at multiple modalities but is best imaged with cardiac MRI. Both CT and MRI adequately image complications related to MI.
